# Identification of Potential New *Aedes aegypti* Juvenile Hormone Inhibitors from N-Acyl Piperidine Derivatives: A Bioinformatics Approach

**DOI:** 10.3390/ijms23179927

**Published:** 2022-09-01

**Authors:** Lúcio R. Lima, Ruan S. Bastos, Elenilze F. B. Ferreira, Rozires P. Leão, Pedro H. F. Araújo, Samuel S. da R. Pita, Humberto F. De Freitas, José M. Espejo-Román, Edla L. V. S. Dos Santos, Ryan da S. Ramos, Williams J. C. Macêdo, Cleydson B. R. Santos

**Affiliations:** 1 Graduate Program in Medicinal Chemistry and Molecular Modeling, Federal University of Pará, Belém 66075-110, PA, Brazil; 2 Laboratory of Modeling and Computational Chemistry, Department of Biological and Health Sciences, Federal University of Amapá, Macapá 68902-280, AP, Brazil; 3 Laboratory of Organic Chemistry and Biochemistry, University of the State of Amapá, Macapá 68900-070, AP, Brazil; 4 Bioinformatics and Molecular Modeling Laboratory, Pharmacy College, Federal University of Bahia, Av. Barão de Jeremoabo, 147, Ondina, Salvador 40170-115, BA, Brazil; 5 Health Department, State University of Feira de Santana, Feira de Santana 44036-900, BA, Brazil; 6 Department of Pharmaceutical and Organic Chemistry, Faculty of Pharmacy, Campus of Cartuja, University of Granada, 18071 Granada, Spain; 7 Laboratory of Molecular Modeling and Simulation System, Federal Rural University of Amazônia, Rua João Pessoa, 121, Capanema 68700-030, PA, Brazil

**Keywords:** juvenile hormone, piperidine derivatives, pharmacophore, ADME/Tox, docking, molecular dynamics, MMPBSA

## Abstract

*Aedes aegypti* mosquitoes transmit several human pathogens that cause millions of deaths worldwide, mainly in Latin America. The indiscriminate use of insecticides has resulted in the development of species resistance to some such compounds. Piperidine, a natural alkaloid isolated from *Piper nigrum*, has been used as a hit compound due to its larvicidal activity against *Aedes aegypti*. In the present study, piperidine derivatives were studied through in silico methods: pharmacophoric evaluation (PharmaGist), pharmacophoric virtual screening (Pharmit), ADME/Tox prediction (Preadmet/Derek 10.0^®^), docking calculations (AutoDock 4.2) and molecular dynamics (MD) simulation on GROMACS-5.1.4. MP-416 and MP-073 molecules exhibiting ΔG binding (MMPBSA −265.95 ± 1.32 kJ/mol and −124.412 ± 1.08 kJ/mol, respectively) and comparable to *holo* (ΔG binding = −216.21 ± 0.97) and pyriproxyfen (a well-known larvicidal, ΔG binding= −435.95 ± 2.06 kJ/mol). Considering future in vivo assays, we elaborated the theoretical synthetic route and made predictions of the synthetic accessibility (SA) (SwissADME), lipophilicity and water solubility (SwissADME) of the promising compounds identified in the present study. Our in silico results show that MP-416 and MP-073 molecules could be potent insecticides against the *Aedes aegypti* mosquitoes.

## 1. Introduction

*Aedes aegypti* (*Diptera*: *Culicidae*) is the main vector of viral diseases such as yellow fever, dengue, chikungunya and Zika in tropical and subtropical areas around the globe [1,2,3]. Arboviruses transmitted by this vector are responsible for hundreds of millions of global infections annually, resulting in considerable socioeconomic and health impacts and representing a major challenge for emerging countries located in regions with large mosquito populations [4]. According to the World Health Organization (WHO) and the Pan American Health Organization (PAHO), dengue cases in the Americas exceeded three million in 2019, surpassing the largest historical epidemic in 2015 (2.35 million cases), with the number of cases in Brazil reaching 2,241,974 (70% of total cases in the region, the highest incidence) [5].

Pharmacological treatments for diseases transmitted by *Aedes aegypti* are palliative; the susceptibility to different types of viruses means that the use of such medicinal alternatives does not add much utility, and access to vaccines is not guaranteed to everyone who need them [6]. Barrier methods have been used to effectively decrease transmission of disease via mosquitoes, involving a set of strategies that incorporate methods to combat the vector and/or individual protection through the use of substances with repellent properties [7].

According to the Resistance Insecticide Action Committee (IRAC) [8], insecticidal compounds have multiple modes of action on various biological targets. Among currently available vector-combating methods, chemical control stands out, as it involves the use of compounds (larvicides and adulticides) that are toxic to insects, inhibiting or blocking proteins, enzymes or channels and promoting the eventual death of the insect [9]. 

**Figure 1:**
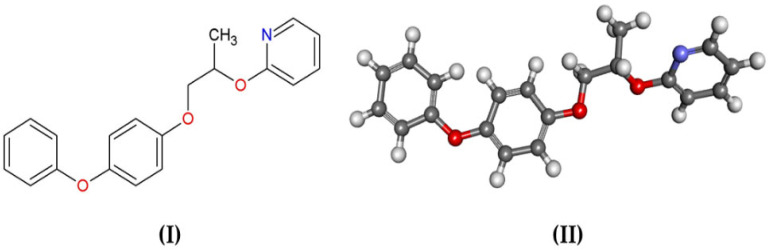
(**I**) 2D structure and (**II**) 3D structure of pyriproxyfen (2-[1-methyl-2-(4 phenoxyphenoxy) ethoxy]pyridine, CAS 5737-68-1).

Juvenile hormone (JHIII) mimetic compounds, represented by pyriproxyfen (Figure 1), are a promising approach in insecticide development, as they regulate the growth process, interrupting endocrine processes, such as metamorphosis [10,11].

The crystallographic structure of *Ae. aegypti* juvenile hormone-binding protein (AagJHBP) is deposited in the Protein Data Bank (https://www.rcsb.org/, accessed on 26 September 2020) under accession number PDB ID 5V13 [12].

The indiscriminate use of insecticidal compounds promotes mosquito resistance, as represented by carbamate larvicides [13]. A recent study showed the effectiveness of pyriproxyfen against *Aedes aegypti* [14]; however, another study reported its species resistance [15].

The piperidine ring is present in natural alkaloids, mainly in the *Piperaceae* family (*Piper chaba*, *Piper guineense*, *Piper longum* and *Piper nigrum*) [16]. Pridgeon et al. [17] synthesized 33 piperidine derivatives (methyl, ethyl and benzyl) and analyzed their toxicity against *Ae. aegypti* female adults through structure–activity relationship (SAR). Later, Doucet and colleagues [18] found that the biological activity associated with one derivative was incorrectly reported. This indicates the need for computational chemical analysis of this class of compounds.

**Figure 2:**
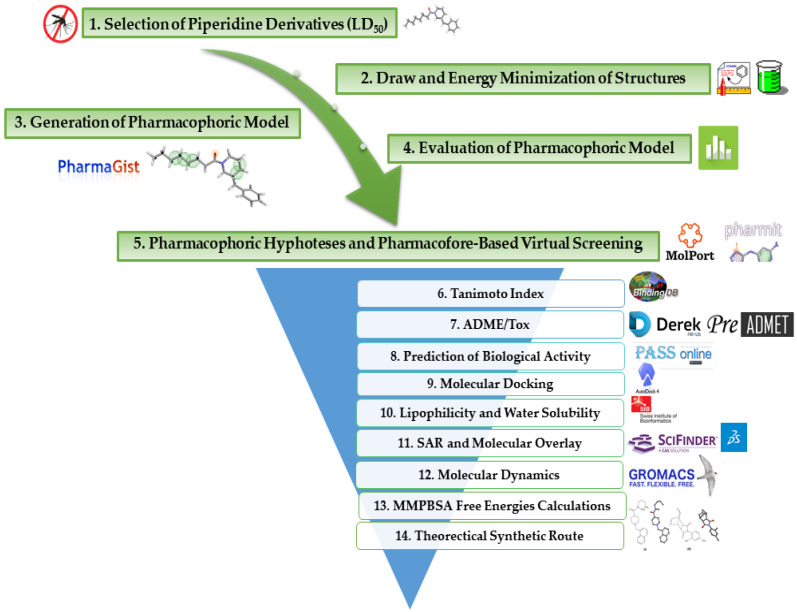
Flow chart of methodological steps employed in this study.

In this research, we searched for new molecules with potential insecticidal activity against the *Aedes aegypti* mosquito using virtual screening approaches. We employed n-acyl piperidine compounds with known biological activity (LD_50_) for pharmacophoric analysis to search for new molecules in the MolPort database, followed by Tanimoto analysis, pharmacokinetic and toxicologic property predictions (ADMET), biological activity predictions on PASS, docking, molecular dynamics simulations and theoretical synthetic route planning for promising compounds. Figure 2 describes the main methodological steps developed in this study.

## 2. Results and Discussion

### 2.1. Structure Selection

N-acyl piperidine compounds were selected based on LD_50_ values described by Doucet [18]. Thus, 30 (thirty) structures were selected, see Table 1, and the compound with the lowest LD_50_ (10.23 mM/mosquito) was selected as the pivot compound (Table 1).

Biological activity values (here, LD_50_) were converted into potency (pLD_50_); this type of parameterization is common in QSAR studies (quantitative structure–activity relationship). With respect to pLD_50_, the most active compounds to have the highest values, decreasing the numerical variation of certain intervals of biological activity data (LD_50_), promoting a normal distribution within the dataset [19,20,21].

### 2.2. Energy Minimization of Structures

The molecules were energy-minimized using HyperChem version 7.51 (Hypercube, Inc., St. Gainesville, FL, USA) software [22] with the MM+ force field. Then, these low-energy conformations were employed on the subsequent steps.

### 2.3. Pharmacophoric Modeling

**Figure 3:**
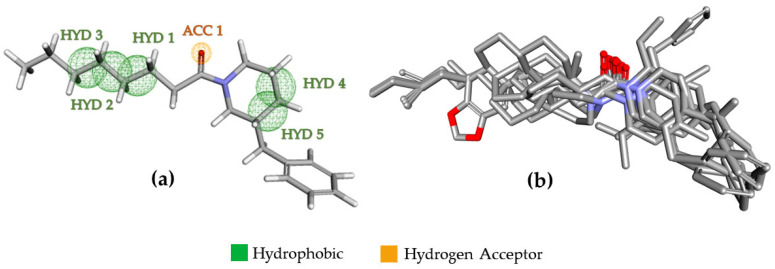
Pharmacophoric model generated by the Pharmagist server [23] (https://bioinfo3d.cs.tau.ac.il/PharmaGist/php.php, accessed on 7 October 2019). (**a**) Pharmacophoric elements located on the pivot structure: one hydrogen bond acceptor (Acc) and five hydrophobic centers (Hyd). (**b**) Alignment of n-acyl compounds (n = 30). Figures (**a**) were taken from the online Pharmit platform, and (**b**) was created by Discovery^®^ Studio Visualizer. In red color, oxygen atoms and nitrogen atoms in blue color.

The pharmacophoric evaluations were performed on the Pharmagist server (https://bioinfo3d.cs.tau.ac.il/PharmaGist/php.php, accessed on 7 October 2019) [23]. The pivot structure (1, Table 1) was aligned to that of other n-acyl piperidines derivatives (Table 1). The best alignment score was 17.234 (n = 30), with six pharmacophoric elements: one hydrogen bond acceptor (Acc) and five hydrophobic centers (Hyd) (Figure 3, Table S1—Supplementary Material).

### 2.4. Pharmacophoric Model Evaluation

From the Pharmagist server [23], we built a matrix describing some pharmacophoric features related to biological activity (pLD_50_ = −logLD_50_) (Table S2—Supplementary Material) and calculated the Pearson correlation coefficients between them: number of atoms (ATM); spatial characteristics (SF); and aromatic (ARO), hydrophobic (HYD) and hydrogen bond acceptors (ACC) (Table 2).

The correlation coefficient varies between positive and negative values among pharmacophoric characteristics (Table 2). There is high correlation between HYD and SF (0.90) and between ATM-SF (0.85), moderate correlation between HYD and ATM (0.68) and between ACC and ARO (0.62), lower correlation between ATM and ARO (0.33) and almost no correlation between ARO and SF (0.05). The values with negative correlation showed a moderate correlation between ACC and HYD (−0.59) and weak correlations between HYD and ARO (−0.36), between ACC and SF (−0.25) and between ACC and ATM (−0.24).

**Figure 4:**
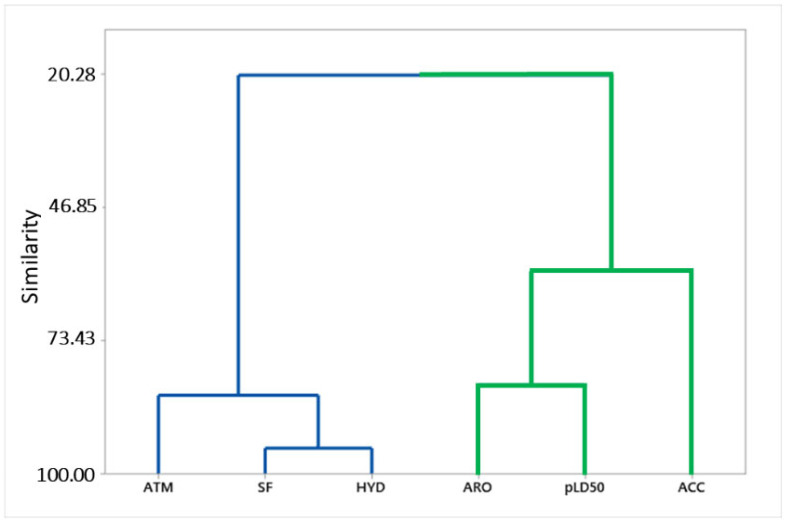
Hierarchical cluster analysis (HCA) of pharmacophoric features related to biological activity (pLD_50_ = −logLD_50_) of n-acyl piperidine compounds. In the green line, properties of the most active structures, and in the blue line the least active.

**Figure 5:**
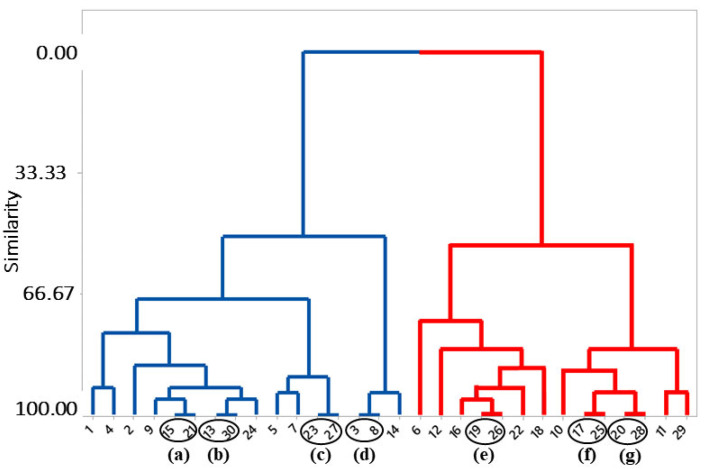
Hierarchical cluster analysis (HCA) of the structures of n-acyl piperidine compounds. (**a**) 15/21; (**b**) 13/30; (**c**) 23/27; (**d**) 3/8; (**e**) 19/26; (**f**) 17/25; (**g**) 20/28. In the blue line, the most active structures of the studied group, and in the red line, the least active according to the pharmacophoric model.

The correlation coefficient with biological activity (pLD_50_) and pharmacophoric properties showed positive results. There is a moderate correlation between pLD_50_ and ARO (0.646) and a lower correlation between pLD_50_ and ATM (0.398), between pLD_50_ and SF (0.277) between pLD_50_ and ACC (0.187) and between pLD_50_ and HYD (0.019). These results suggest that pharmacophoric elements and biological activity vary proportionally to the number of aromatics (ARO), reinforcing the role of these groups with respect to biological activity. In order to further analyze the chemical structure correlations with biological activity, a multivariate statistical analysis technique called hierarchical cluster analysis (HCA) was employed (Figure 4 and Figure 5) [19,21].

Hierarchical cluster analysis (HCA) obtained similar results to those presented in Table 2, and Pearson’s distance was used to build a dendrogram with pharmacophoric characteristics as dependent variables. This analysis confirms the relationship of spatial (SF) and hydrophobic (HYD) characteristics, as well as the number of atoms (ATM) and acceptor (ACC) and aromatic (ARO) characteristics, with biological activity (pLD_50_) (Figure 4). The aromatic (ARO) and biological activity (pLD_50_) characteristics showed a good approximation in the green cluster. The number of atoms (ATM), as well as spatial (SF) and hydrophobic (HYD) properties, exhibited high similarity in the blue cluster.

The HCA method showed similarity between chemical structures, classifying n-aryl piperidine compounds into two active groups according to their pharmacophoric elements (ATM, SF, ARO, HYD and ACC). In the blue cluster contains the 16 most active molecules, and the red cluster includes the 14 least active molecules (Figure 5).

We observed some structures with 100% similarity: 15/21; 13/30; 23/27; 3/8; 19/26; 17/25 and 20/28 (Figure 5a–g) because all molecules have the same pharmacophoric characteristics (ATM, SF, ARO, HYD and ACC) and structural arrangements. To understand this similarity, we analyzed steric and electrostatic molecular field overlaps in Biovia Discovery Studio Visualizer (Dassault Systémes, Vélizy-Villacoublay, France) (Table 3). It is well-known that steric and electrostatic forces play a fundamental role in the biological activity of molecules, as they directly influence their conformations [24].

Structures 15 and 21 showed the best similarity results, with 100% steric contribution, exhibiting 0.9277 (92.77%) similarity in the steric fields. With respect to the 100% electrostatic contribution, structures 23 and 27 presented 0.9173 (97.73%) similarity in the electrostatic fields. Interestingly, these structures are part of the most active group of molecules in the set.

The lowest molecular overlap values among these similar structures are those of molecules 3 and 8, which exhibited a similarity of 0.7602 (76.02%) for 100% steric contribution and 0.7268 (72.68%) for 100% electrostatic contribution. These data show that these structures listed in this analysis have a significant degree of similarity, corroborating the results of the HCA dendrogram (Figure 5). The structures that are grouped with 100% similarity form clusters with other structures, also with significant levels of similarity; for example, structures 15–21 form a cluster with structure 9, structures 13–30 with 24 and structures 3–8 with 14.

Despite having similar steric and electrostatic fields, these molecules have structural characteristics that visually differ from one another (Figure S2—Supplementary Material). Similar to the 30 n-acyl piperidine derivatives presented in this study, these structures also share a common group, i.e., piperidine ring linking, through a pair of unpaired electrons of its nitrogen atom (N), to a carbonyl group.

### 2.5. Pharmacophoric Hypotheses and Pharmacophore-Based Virtual Screening

In our search for new molecules through ligand virtual screening, maximum and minimum values of physicochemical properties were used as filters (Table S3—Supplementary Material).

Ferreira and colleagues [21] cited that pharmacophoric characteristics were used in varying combinations for the construction of hypotheses using Equation (1) below:

(1) $$C_{p,n}=\frac{n!}{p!\left(n-p\right)!}$$

where *C* = the number of combinations, *p* = the type of model (*p* ≠ 0, *p* = 1, *p* = 2, …, *p* = ∞) and *n* = the number of variables in the pharmacophoric model. Five variables were considered (Hyd 1, 2, 3, 4 and 5) and analyzed by simple combination without repetition. The 5 possible combinations (pharmacophoric hypotheses) obtained were submitted to the PHARMIT platform (http://pharmit.csb.pitt.edu/search.html [25], accessed on 11 November 2019) to search for new molecules in order to obtain as many hits as possible in the MolPort^®^ database (https://www.molport.com/, accessed on 11 November 2019), as shown in Table 4.

### 2.6. Tanimoto Similarity

A total of 1312 structures were selected in pharmacophore-based virtual screening. Some compounds that presented a Tanimoto similarity index ≤ 0.35 were eliminated, and the 145 molecules most similar to the pivot (Table 1) were selected for pharmacokinetic and toxicological predictions.

### 2.7. Prediction of Toxicological and Pharmacokinetic Properties

A total of 145 structures were analyzed on DEREK [26] based on toxicological alerts (Table S4—Supplementary Material). Molecules that presented some warnings (hepatotoxicity, carcinogenicity, skin sensitivity, teratogenicity, hERG channel inhibition, photoallergenicity, mutagenicity, peroxisome proliferation and phospholipidosis for humans, rats and mice) [11,21] were eliminated from our dataset, resulting in 79 molecules.

Subsequently, these structures were submitted to the Preadmet server (https://preadmet.webservice.bmdrc.org/ [27], accessed on 11 January 2020), which predicts carcinogenicity as “positive”, if there are no carcinogenic features and “negative” otherwise [28]. Among the control compounds, the pivot molecule (the most active of the studied series) showed carcinogenic properties, whereas 12 of the 79 molecules in the DEREK analysis did not show carcinogenic properties, indicating the toxic potential of these compounds (Table 5).

Pharmacokinetic analysis was performed with the 12 compounds that did not show toxicity in the previous step (Table 6). Prediction of intestinal abortion is one of the main goals in the selection of molecules with potential biological activity [29]. The human intestinal absorption (HIA) percentage (Table 6) was greater than 70% in all molecules, with the lowest value of 98.14%. The permeability of Caco-2 and MDCK (Mandin–Darby canine kidney) cells was also investigated, values > 500 nm s^−1^ indicating satisfactory permeability and values < 25 nm s^−1^ indicating weak permeability [21,30,31]. Some molecules had values < 25 nm s^−1^ for cell permeability for both CACO-2 and MDCK, whereas others showed unsatisfactory results for both permeability models (Table 6). Skin permeability (P_skin_) is a consequence of phenyl acylation of the skin protective esters [29]. The permeability values of new molecules varied between −1 and −4 cm h^−1^, i.e., between the acceptable range (−1 to −8) and the impermeable range [11].

The plasma protein binding (PPB) percentage can influence the effectiveness of bioactive compounds, with a reference value of >90% [28]. Most of the structures showed a percentage of PPB greater than 90%, with a minority showing data very close to the parameters described in the literature. Blood–brain barrier penetration (C_brain_/C_blood_) is related to the action of compounds in the central nervous system [29], with a reference value of <1, as any other value indicates that the compound is highly concentrated in the CNS, causing adverse effects [11,30]. Most of the structures align with the reference values (Table 6).

### 2.8. Molecular Docking Simulation

**Figure 6:**
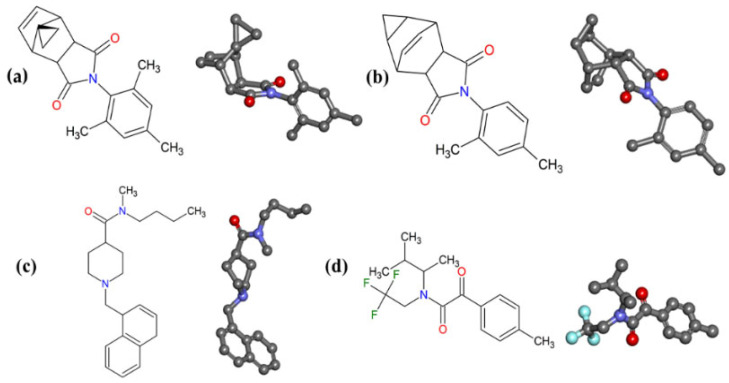
Chemical structures with the most accurate pharmacokinetic and toxicological results from the MolPort^®^ database. (**a**) MP-779; (**b**) MP-073; (**c**) MP-416; (**d**) MP-112.

The four (4) structures with the most accurate toxicological and pharmacokinetic predictions (Figure 6) were used for molecular docking simulations, with the objective of evaluating pharmacodynamic aspects of these structures at the active site of the biological receptor. The active site was determined by ligand crystallographic pose (JHIII, PDB ID: 5V13). The redocking value was JHIII RMSD = 1.835 Å (Figure S3—Supplementary Material). RMSD values ≤ 2 Å validated redocking [31].

Kim and colleagues (2017) [12] described the active site interactions in the juvenile hormone-binding protein of the *Aedes aegypti* mosquito (AagJHBP) (PDB ID: 5V13) with only one conventional hydrogen bond (Tyr129) in the protein structure. Hydrophobic interactions are located around the α-helix (Ser30-Ala38, Arg45-Glu51, Val60-Gln71, Phe123-Leu130, Val132-Arg136, Leu138-Arg143 and Val280-Trp286) and β-sheet (Pro52-Pro55, Tyr72-Val73, Thr144-Val145 and Arg276-Gln279).

**Figure 7:**
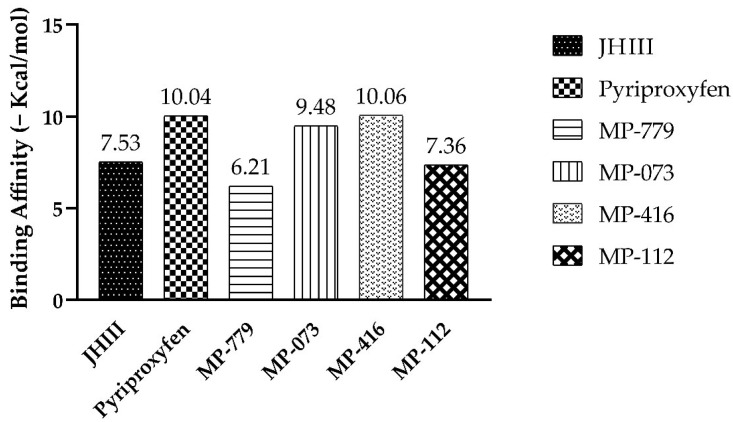
Binding-affinity energies for complexed ligands at the active site of *Aedes aegypti* juvenile hormone-binding protein (AagJHBP, PDB ID: 5V13).

In order to evaluate the binding affinity (ΔG) of the promising compounds at the AagJHBP active site, we compared all values. Only one molecule showed a ΔG value greater than pyriproxyfen and juvenile hormone (JHIII), as shown in Figure 7.

**Figure 8:**
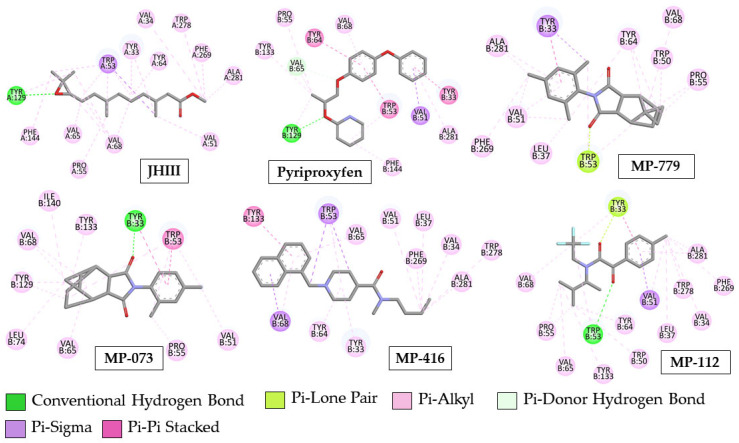
Interactions of the tested molecules (MP-779, MP-073, MP-416 and MP-112) with the juvenile hormone protein of *Aedes aegypti* (JH) (PDB ID 5V13).

Among the compounds docked in AagJHBP (PDB ID: 5V13), MP-416 presented with the best binding affinity (ΔG = −10.06 kcal/mol) surpassing the binding affinities of the crystallographic ligand (JHIII) and control compound (Pyriproxyfen) (Figure 7). Despite not performing any conventional hydrogen bonding like JHIII and pyriproxyfen, the MP-416 molecule performed only hydrophobic interactions at the AagJHBP active site with amino acid residues like JHIII (Figure 8).

The commercial compound pyriproxyfen showed the second highest binding affinity (ΔG = −10.04 kcal/mol), with hydrophobic interactions and hydrogen bonds in common with JHIII (Figure 8). Compound MP-073 also showed significant binding affinity (ΔG = −9.48 kcal/mol) but lower than that of JHIII and pyriproxyfen. This compound exhibited hydrophobic interactions with most of the amino acid residues that interacted with JHIII (Figure 8). 

Compounds MP-779 and MP-112 showed binding affinity below the complexed ligand (JHIII) at the active site, exhibiting ΔG values of −6.21 and −7.36 kcal/mol, respectively. Although these compounds (MP-779 and MP-112) showed hydrophobic interactions in common with the complexed ligand, JHIII, they also showed unusual hydrophobic interactions with important amino acid residues of the active site (Figure 8). 

Molecules MP-416 and MP-073 exhibited favorable interactions, as represented by their binding affinity (−10.06 kcal·mol^−1^ and −9.48 kcal·mol^−1^), compared with the two reference compounds (JHII and pyriproxyfen).

Docking studies of MP-416 (the highest binding affinity) showed Pi–Alkyl interactions with Tyr33, Val34, Val51, Tyr64, Val65, Val68, Phe269, Trp278 and Ala 281 residues and Pi–Sigma interactions with Trp53, corroborating the data presented by Kim and colleagues [12]. In addition, MP-416 had other Pi-Alkyl interactions with Leu37, Pi–Sigma interactions with Val68 and Pi–Pi stacked interactions with Tyr133 (Figure 8).

MP-073 had hydrophobic interactions (Pi–Alkyl) with Val51, Trp53, Pro55, Val65, Val68 and Tyr129, corroborating the interactions with complexed ligand JHIII [12], as well as Leu74, Tyr133 and Ile140 residues. Other interactions included Pi–Pi stack with Trp53 and hydrogen bond interactions with Tyr33 (Figure 8). 

MP-112 had Pi–Alkyl interactions with Tyr33, Val34, Val51, Trp53, Pro55, Tyr64, Val65, Val68, Phe269, Trp278 and Ala281, as crystallographic ligand [12], as well as Pi–Alkyl interactions with Leu37, Trp50 and Tyr133, Pi–Lone pair interactions with Tyr33 and hydrogen bond interactions with Trp53, see Figure 8. 

MP-779 (lowest binding affinity compound) had Pi–Alkyl interactions with Val51, Trp53, Pro55, Tyr64, Val68, Phe269 and Ala281, as well as the JHIII ligand, Pi–Alkyl interactions with Trp50 and Leu37, Pi–Lone pair interactions with Tyr53 and Pi–Sigma interactions with Tyr33 (Figure 8). All this information is presented in Table S5—Supplementary Material.

Responsible for the growth, development, metamorphosis and reproduction of the insect, the juvenile hormone is secreted by specific endocrine glands. This receptor, which comprises a large group of insects (not only *Aedes aegypti*), has been the target of study in the development neurotoxic insecticides and pesticides through classical bioassays to measure the agonist activity of this hormone [32].

With these docking results, we proposed that these compounds can bind to the active sites of *Aedes aegypti* juvenile hormone-binding protein (AagJHBP), and MP-416 and MP-073 obtained the best binding affinity (Figure 8).

### 2.9. Structure–Activity Relationship (SAR) and Molecular Overlay of Promising Molecules

The identified promising molecules (Figure 6) were searched on the Scifinder^®^ online server (https://scifinder.cas.org/, accessed on 5 August 2020) with the aim of obtaining information about molecules with biological activity. 

The only molecule that returned experimental data in the search was MP-779, as this molecule was patented and used for the preparation of fused cyclic succinimide compounds and their analogues as modulators of nuclear hormone receptor function [33].

The molecules (a) MP-779 and (b) MP-073 share the pyrrolidine-2,5-dione group (C_4_H_4_NO_2_) in common. This group is present in derivatives isolated from *Tribulus terrestris* fruits. According to a phytochemical study, the fruits of this plant species have several chemical constituents with important medicinal aspects, in addition to presenting larvicidal properties [34], whereas the molecule (c) MP-416 has a piperidine group (C_5_H_11_N) in its structure. 

As previously mentioned, experimental data corroborate the larvicidal activity of piperidine-derived molecules (natural and synthetic) in *Aedes aegypti* mosquitoes [17,18]. In molecule (d) MP-112 contains a trifluoromethyl group (CF_3_), a group that can enhance the biological activity of molecules due to its intrinsic properties, such as electronegativity and high lipophilicity. 

The presence of this group in some pesticides is well-recognized [34,35,36]. With the aim of correlating with the virtual screening data, the similarities between the MolPort^®^ molecules and the n-acyl piperidine compounds were analyzed according to their steric and electrostatic molecular field overlaps. 

The structures were overlaid, taking into consideration the percentage of steric contribution at 50%, 70% and 100% of the four promising molecules in relation to the pivot structure and the commercial compound pyriproxyfen (Table 7).

**Figure 9:**
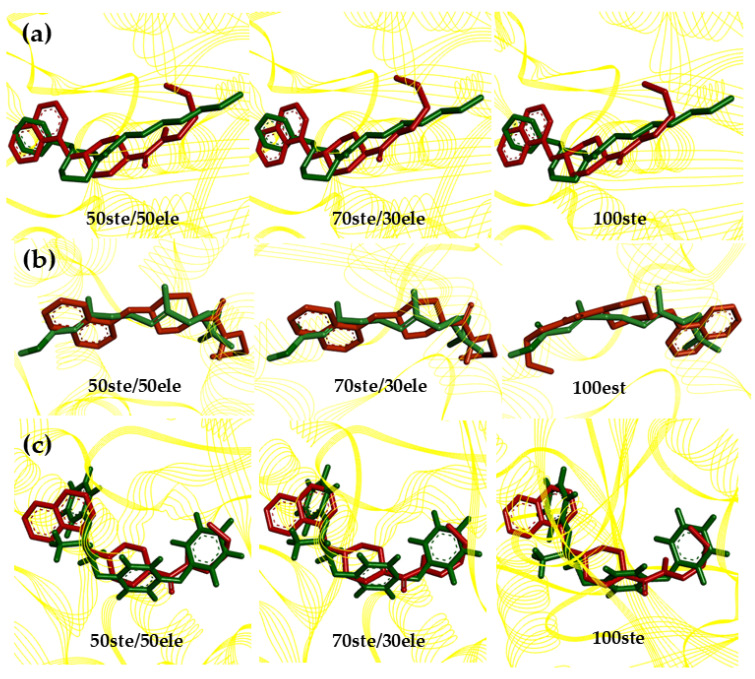
Similarity analysis according to the superposition of the steric fields of the MP-416 molecule (red), the pivot molecule (green, (**a**)), JHIII (green, (**b**)) and pyriproxyfen (green, (**c**)).

In the overlapping results for 50ste (50% steric contribution), the promising molecules showed overlapping similarity values that varied from 32% to 69%. For 70%, the promising molecules showed values ranging from 48% to 70% in terms of steric similarity. Finally, for 100%, the promising molecules presented with variation of 71% to 78%. The best overlap values occurred between the promising molecule MP-416, the pivot molecule, the JHIII ligand and the commercial compound pyriproxyfen (Figure 9).

### 2.10. Molecular Dynamics (MD) Simulations

With the aim of understanding the inhibitory behavior of pyriproxyfen, MP-073, MP-416 and *Aedes aegypti* juvenile hormone-binding protein (AagJHBP, PDB ID: 5V13), we ran 100 ns of molecular dynamics (MD) simulations on GROMACS 5.1.4 (University of Groningen, Groningen, The Netherlands) [37,38]. These data allowed us to compare the time evolution of five systems (apo, holo, pyriproxyfen, MP-073 and MP-416).

**Figure 10:**
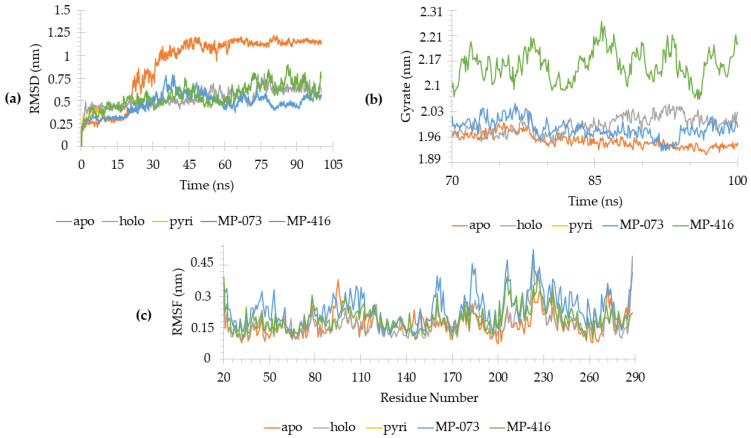
Molecular dynamics (MD) simulation data generated in GROMACS 5.1.4 [37,38,39,40,41,42,43,44] from *Aedes aegypti* juvenile hormone-binding protein (AagJHBP, PDB ID: 5V13) complexes. (**a**) RMSD values (averaged mean ± standard deviation) for each system: apo (1.13 ± 0.04), holo (0.46 ± 0.04), Pyri (0.59 ± 0.09), MP-073 (0.49 ± 0.05) and MP-416 (0.69 ± 0.08). (**b**) Radius of gyration and (**c**) RMSF values for each system.

We first analyzed the stability of these complexes during MD (Figure 10). Root-mean-square deviation (RMSD) showed that all systems equilibrated after 50 ns (Figure 10a), defining our productive phase from 70–100 ns for all simulations for all further analyses. The radius of gyration of AagJHBP as a function of productive time showed that protein stabilized in all systems (Figure 10b), reinforcing the RMSD data (Figure 10a). The fluctuation of the protein structure during the productive phase ensured that a large part of the amino acid residues remained stabilized, taking into account the variation of RMSF (Figure 10c).

**Figure 11:**
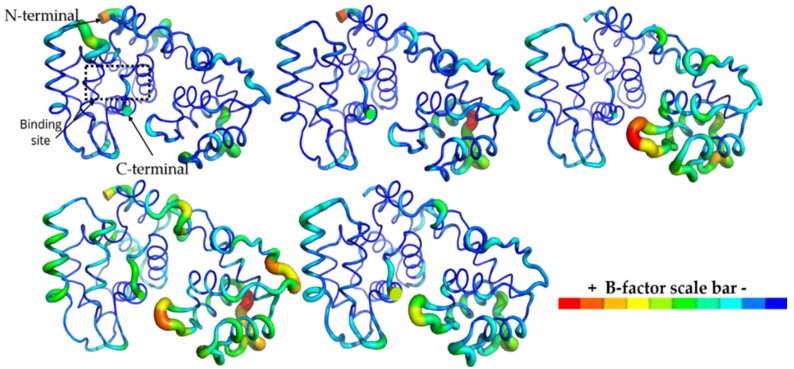
RMSF-3D from *Aedes aegypti* juvenile hormone-binding protein (AagJHBP) structures. The highest B factor is colored red and the lowest, dark blue. The thickness of the protein backbone is proportional to the B factors. This image was generated by educational PyMOL 2.4.1 [45]. From the upper-left corner to the right: apo, holo, Pyri, MP-073 and MP-416.

The 3D root-mean-square fluctuation (RMSF-3D) could reveal which part is disturbed when complexed with ligands. By analyzing these data, we noted that the active site remained stable, despite the ligand binding, and both MP-073 and MP-416 stabilized AagJHBP, similarly to the natural substrate (holo) and pyriproxyfen complexes (Figure 11). These data correlate with the crystallographic structure (AagJHBP, PDB ID: 5V13) of *Aedes aegypti* juvenile hormone-binding protein, with active site residues solved in electron density maps with a resolution of 1.84 Å [12].

Some regions presented with a higher fluctuation in the AagJHBP structure, mostly at loops helices, as well as N and C terminals (Figure 11). AagJHBP crystallography data showed helices and loop regions within binding site residues (Pro55-Val65) and distant residues (Gln90-Glu100).

Our MD data revealed that these helices and loops on the N-terminal domain fluctuated less than those on the C-terminal domain (Figure 11). Previous studies described the N terminal of AagJHBP as the region responsible for the stability of the complex [12]. Based on these results, we analyzed the interaction pattern on AagJHBP and the identified promising compounds compared with the crystallographic ligand (methyl (2E,6E)-9-[(2R)-3,3-dimethyloxiran-2-yl]-3,7-dimethylnona-2,6-dienoate, JHIII).

We also analyzed their secondary structure stability using the DSSP 3.1.4 module [46,47,48] installed on GROMACS 5.1.4 (University of Groningen, Groningen, The Netherlands) [37,38]. All *Aedes aegypti* juvenile hormone-binding protein (AagJHBP) complexes globally maintained a stable secondary structure during our simulation (Figure S4—Supplementary Material).

**Figure 12:**
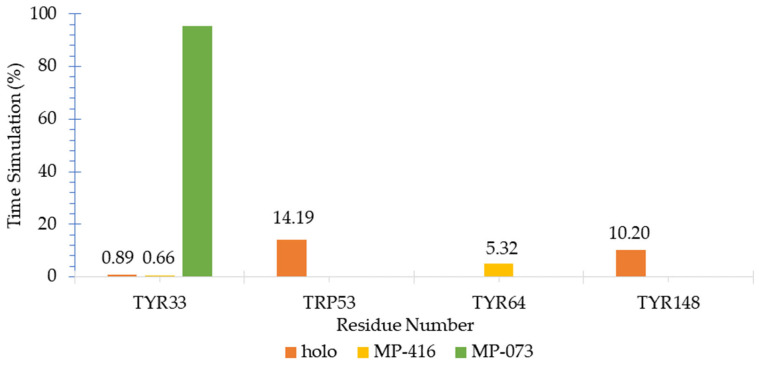
Hydrogen bond stability on *Aedes aegypti* juvenile hormone-binding protein (AagJHBP) complexes calculated by HbMap2Grace for the productive phase.

The hydrogen bond pattern was evaluated using the GROMACS Hbond module [49] and the HbMap2Grace program [50], and the molecular surface area was evaluated with the SurfinMD program [51]. Molecular dynamics (MD) data for hydrogen bond analysis (H-bond) showed that promising compounds (MP-073 and MP-416) had less frequent H-bond interactions than JHII, corroborating the docking data (Figure 12). Because these interaction patterns could favor their inhibitory behavior, we analyzed them in detail.

Our results from the MD productive phase correlate well with the estimated binding energy obtained before docking (Table S5—Supplementary Material), i.e., the ligands with the best docking energies presented with H-bond permanency, suggesting that a hydrogen bond is a favorable interaction for the development of AagJHBP binding compounds [44]. 

The H-bond pattern presented interactions with Tyr33, Trp53, Tyr64 and Tyr148. The holo complex showed hydrogen bonding with residue Tyr33 in a shorter time interval when compared to residues Trp53 and Tyr148. MP-073 showed hydrogen bonding during almost the entire simulation time with the Tyr33 complex. 

The MP-416 complex presented hydrogen bonding in a short time interval with the Tyr33 residue and in a longer simulation interval with the Tyr64 residue; the latter plays an important role in the stabilization of the system [12].

**Figure 13:**
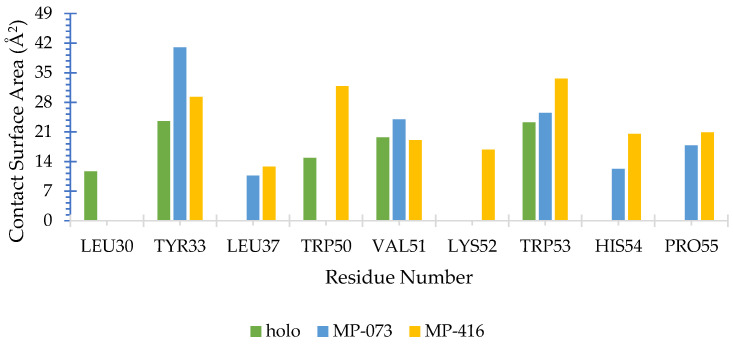
Surface molecular area (Å^2^) of *Aedes aegypti* juvenile hormone-binding protein (AagJHBP) complexes calculated by SurfinMD for the productive phase.

We also calculated the atomic contacts involving AagJHBP and the identified promising compounds (Figure 13). The contact surface area revealed interactions with the nonpolar residues Val51 and Trp53 and with the polar residue Tyr33 present in the three complexes (Holo, MP-073 and MP-416), corroborating data reported in the literature [12]. The Holo complex showed an additional interaction with non-polar residue Trp50, whereas the MP-073 complex showed additional interactions with Leu-37 and His-54. The MP-416 complex showed the highest number of additional interactions, with residues Trp50, Lys52, His54 and Pro55.

#### MMPBSA Free Energy Calculation

**Figure 14:**
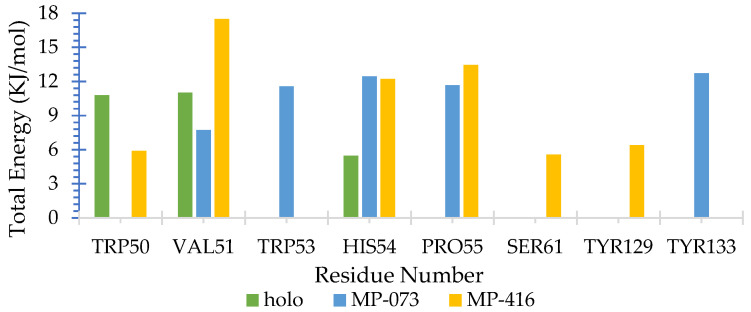
Residue contributions to the binding energy of *Aedes aegypti* juvenile hormone-binding protein (AagJHBP) complexes. The main residues with energy interaction (ΔE_binding_ > ±5 kJ/mol) are highlighted.

We also calculated the binding free energy of all AagJHBP complexes through MM-PBSA methods. The binding energy (ΔE_binding_) calculated by solvent accessible surface area showed that all compounds interacted favorably with AagJHBP (Table 8). Because these values are directly correlated with interacting protein residues, we decided to discriminate which amino acids presented better contacts with ligands. For these residue decomposition energy analyses, we selected residues near the ligand (<5 Å) during the MD simulation and that participated actively in complex stabilization (ΔE_binding_ > ±5 kJ/mol), as shown in Figure 14.

We noted that residues did not interact favorably with ligands in all complexes (positive ΔE_binding_ value). The residues that presented contributions in the three complexes (holo, MP-073 and MP-416) were Val51 and His54. The residues Trp53 and Tyr133 presented contributions only to the MP-073 complex, with Ser61 and Tyr129 contributing to the MP-416 complex. Among the residues shown in Figure 14, Val51, Trp53 and Trp129 constitute part of the active site described by Kim et al. [12].

### 2.11. Synthetic Accessibility and Theoretical Synthetic Route of Promising Compounds

#### 2.11.1. Synthetic Accessibility via SwissADME Webserver

The MP-073 structure presented an SA score of 3.85 (Table 9), whereas the MP-416 structure presented a score of 2.12. In comparison to the control compounds (pivot, JHIII and pyriproxyfen), the SA values were close, ranging between 2.12 and 3.85. The MP-416 compound is the easiest to synthesize compared to the other compounds. Both structures (MP-073 and MP-416) were considered easy to synthesize, considering the parameters reported in the literature [52].

#### 2.11.2. Theoretical Synthetic Route

The molecules MP-416 and MP-073 proved to be the most promising of the investigated compounds, with easy synthesis; therefore, we elaborated a theoretical synthesis route for these compounds with a view to future biological tests (in vitro and in vivo). 

**Figure 15:**
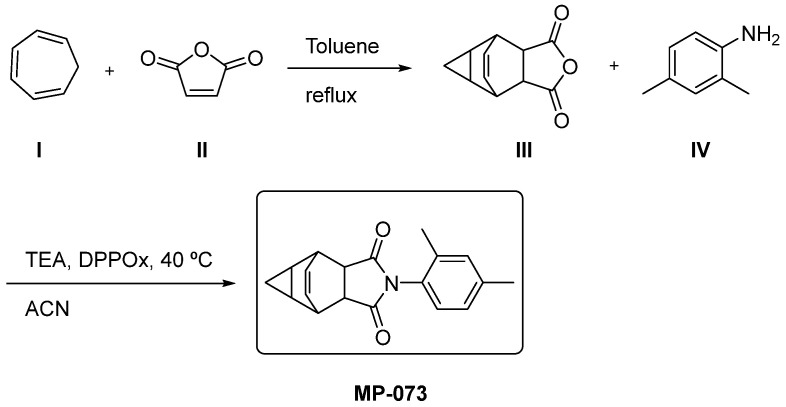
Theoretical synthetic route for the preparation of compound MP-073. Starting materials I, II and IV are commercially available.

We present a synthetic route of compound MP-073 based on the preparation of benzofurandione III as a key intermediate (Figure 15). Substrate III can be formed using cycloheptatriene I and maleic anhydride II as starting materials in refluxing of toluene by a Diels–Alder reaction, as reported by B. A. Selivanov et al. [53]. Subsequently, a dehydrating condensation [54] between III and aniline IV using triethylamine (TEA) and diphenyl 2-oxo-3 oxazolinyl phosphonate (DPPOx) in acetonitrile (ACN) will yield indoledione MP-073.

**Figure 16:**
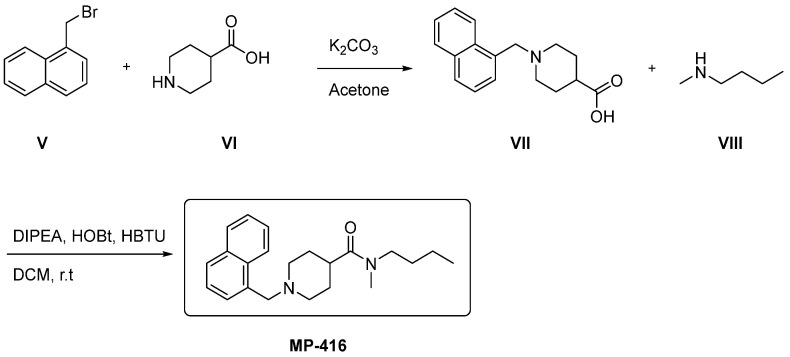
Theoretical synthetic route for the preparation of compound MP-416. Starting materials V, VI and VIII are commercially available.

Compound MP-416 can be obtained in two synthetic steps (Figure 16). First, an alkylation reaction of naphthalene V with carboxylic acid VI using potassium carbonate (K_2_CO_3_) as a base in acetone will provide intermediate VII [55]. Finally, the desired carboxamide MP-416 will be provided by the reaction between carboxylic acid VII and amine VIII using hydroxybenzotriazole (HOBT), N,N-diisopropylethylamine (DIPEA) as a base, 2-(1H-benzotriazol-1-yl)-1,1,3,3 tetramethyluronium hexafluorophosphate (HBTU) as a coupling agent and dichloromethane (DCM) as solvent [56].

### 2.12. Lipophilicity and Water Solubility via SwissADME Webserver

**Figure 17:**
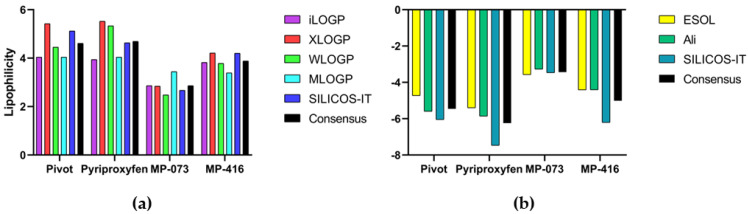
Prediction of (**a**) LogP and (**b**) LogS values of control molecules (pivot and pyriproxyfen) and promising compounds (MP-779, MP-073, MP-416 and MP-112).

After obtaining the molecules via synthesis, we believe that this methodological proposal will aid medicinal chemists in the elaboration of solutions based on their solubility, considering future in vivo assays, in order to validate the computational methods used. The pivot and commercial compounds (pyriproxyfen) showed the highest heats of LogP_o/w_ consensual (black column) (Figure 17). These compounds exhibit low solubility in water, requiring organic compounds for solubilization. Pyriproxyfen has been reported to be soluble in ethanol, DMSO and dimethylformamide (DMF) [57]. Among the promising molecules investigated in the present study, the structure MP-416 had the highest consensus value of LogP_o/w_. This can be attributed to the methylnaphthalene group present at the bottom of its chemical structure, which is an aromatic and hydrophobic group. The structure of MP-773 comprises important chemical groups, such as dimethyl and trimethylphenyl, in addition to cyclic hydrocarbon groups, which reflects the LogP values. In this study, the predicted LogP_o/w_ values were found to be positive in the range of +2.08 to +6.93 (Table S6—Supplementary Materials, Figure 17), suggesting that all molecules are highly lipophilic.

The LogS values of the compounds for the ESOL method ranged between −3.58 and −6.16; for the Ali method, between −3.28 and −9.19; and for the SILICO-IT method between, −3.47 and −7.47 (Table S7—Supplementary Material, Figure 17). By consensus, these data suggest that most compounds are moderately or poorly soluble in water, as they are in the range −4 to −6, which suggests that their solubilization in water is only possible with organic solvents, as is the case of the pivot commercial compound Pypriproxyfen. Only molecule MP-073 showed good water solubility according to all three estimation methods, with values in the range of −2 to −4.

## 3. Materials and Methods

### 3.1. Selection of Structures

The compounds (Figure S1—Supplementary Material) were selected based on studies by Doucet et al. [18] wherein the authors used QSAR methods to predict the toxicity of piperidine derivatives against *Aedes aegypti*. Based on the LD_50_ values, we selected the most active compounds among these derivatives.

### 3.2. Energy Minimization of Selected Structures

The structures were drawn with ACD/ChemSketch (Advanced Chemistry Development, Inc., Toronto, Ontario, Canada) software [58], and their energies were minimized with HyperChem version 7.51 (Hypercube, Inc., St. Gainesville, FL, USA) [22]. The force field used was MM+ (molecular mechanics), following the methodological strategy proposed by Costa et al. [20].

### 3.3. Pharmacophoric Modeling

After optimization, the structures were opened in Biovia Discovery Studio Visualizer (Dassault Systémes, Vélizy-Villacoublay, France) [59]. These molecules were saved in a single file to obtain pharmacophoric hypotheses through the Pharmagist online server (http://bioinfo3d.cs.tau.ac.il/PharmaGist/, accessed on 7 October 2019). This tool generates 3D structures from aligned features, facilitating the search for candidates based on pharmacophores [20].

Determination of the pharmacophoric hypotheses were used for virtual screening (VS) based on studies by Ferreira et al. [21] and Cruz et al. [28] with the aim of identifying compounds from commercial databases of chemical structures with desirable pharmacological properties, resulting in an increased probability of binding to a particular investigated molecular target.

### 3.4. Pharmacophoric Model Evaluation

Using the data from the descriptors provided by Pharmagist, a matrix containing 6 descriptors was built for Pearson correlation in order to determine the degree of linear relationship between the variables. Pearson’s correlation coefficient (p) has a dimensionless value that comprises the numerical range from −1 to +1. When the correlation coefficient is equal to zero, there is no linear relationship between the variables in question. Values greater than 0.2 indicate a weak correlation, values greater than 0.4 indicate a moderate correlation and values between 0.7 and 0.9 indicate a strong correlation. A perfect correlation is confirmed by a coefficient value of −1 or +1 [21].

With the aim of verifying the relationship between the variables in the pharmacophoric model, hierarchical cluster analysis (HCA) can show the similarity or difference between descriptors individually using the Pearson correlation and distance methods [60,61]. Minitab^®^ 19 (Minitab LCC, State College, PA, USA) software [62] was used for all statistical analyses in the present study.

### 3.5. Pharmacophoric-Based Virtual Screening

The best selected pharmacophoric hypothesis selected were used to study TV with the Pharmit^®^ platform (http://pharmit.csb.pitt.edu, accessed on 11 November 2019), an online tool suitable for virtual screening through a large database of data, offering specific information based on pharmacophores as well as the spatial arrangement of interaction characteristics and molecular shape [25]. In this project, compounds from the MolPort^®^ database (https://www.molport.com/, accessed on 11 November 2019) were selected based on the filter of maximum and minimum values of the studied structures according to the Tice Rule [63]: 150 ≥ MW ≤ 500; 0 ≥ logP ≤ 6.5; HBD ≤ 2; 1 ≥ HBA ≤ 8 and RotB ≤ 12. The values used in the filter were obtained using the online servers Protox II (http://tox.charite.de/protox_II/, accessed on 11 November 2019) and Molinspiration (https://www.molinspiration.com/, accessed on 11 November 2019) in order to proceed with the other stages of virtual screening.

### 3.6. Tanimoto Similarity

The structures obtained from the Molport^®^ database were selected according to Tanimoto similarity through the BindingDB online server (https://www.bindingdb.org/bind/index.jsp, accessed on 5 December 2019), wherein the searched molecules are ranked in order of similarity to the pivot compound [64].

The Tanimoto similarity coefficient (Equation (2)) is a value that varies between 0 and 1, representing the similarity between two compounds based on the fingerprint bits (molecular fragments) between them; the higher the value, the higher the similarity.

(2) $$Tanimoto\;Coefficient=\frac{c}{\left(a+b-c\right)}$$

where fingerprints of two compounds, A and B, (*a*) corresponds to the number of bits in A, whereas *b* corresponds to the number of bits in compound B and *c* corresponds to the number of common bits between compounds A and B [65]. Molecules with a similarity index greater than 0.35 were selected for pharmacokinetic and toxicological predictions.

### 3.7. Prediction of Toxicological and Pharmacokinetic Properties 

The toxicity profiles of the searched compounds were evaluated using Derek Nexus 10.0.2 (Lhasa Limited, Leeds, UK) software [66] based on studies by Ferreira et al. and Ramos et al. Derek (Deductive Estimation of Risk from Existing Knowledge) is a software based on knowledge and biological tests with the objective of qualitatively predicting the toxicity of chemical compounds [11,21].

The biological activity (LD_50_ = mg kg^−1^) and toxicity class properties used in this study were predicted by the Protox II online server [67] (http://tox.charite.de/protox_II/index.php?site=home, accessed on 11 November 2019). The predictions are made based on the similarities of the functional groups of the studied structures, with data from structures previously validated in vitro and in vivo.

Preadmet (https://preadmet.bmdrc.kr/, accessed on 11 January 2020) is an online server used to predict pharmacokinetic and toxicological properties based on *in silico* methods. In this server, pharmacokinetic properties are calculated, such as human intestinal absorption (HIA), in vitro Caco-2 permeability (PCaco-2), skin permeability (P_Skin_), binding to plasma proteins (PPB) and penetration of the blood–brain barrier (C_Brain_/C_Blood_), in addition to toxicological descriptors, such as carcinogenicity (mouse and rat) and mutagenicity (Test and Ames). Preadmet was used according to the methodology described by Cruz et al. [28].

### 3.8. Molecular Docking

Molecular docking structures were prepared using Biovia Discovery Studio Visualizer (Dassault Systémes, Vélizy-Villacoublay, France) [59]. The molecular targets used in this study were juvenile hormone-binding protein from the *Aedes aegypti* vector (AagJHBP), obtained from the Protein Data Bank database (PDB) (https://www.rcsb.org/, accessed on 26 September 2020) [68] with the respective PDB ID: 5V13 [12]. The docking methodology was validated in a redocking study, whereby the crystallographic ligands themselves were submitted to the docking process with AutoDockTools 1.5.6 (CCSB, La Jolla, CA, USA) software [69] to calculate the RMSD (root-mean-square deviation) values of the crystallographic pose of the ligands compared to the computational pose.

The x, y and z coordinates of the active site of each target were selected, as shown in Table 10. Molecular docking was performed according to the protocol described by Bastos et al. [70] and Rocha et al. [71], with the addition of hydrogens and Gasteiger partial charges and mixing with the non-polar hydrogens. The parameter adopted for the present study was AD4, with the Lamarckian G4 algorithm. The simulation was submitted to 100 runs with 150 populations and long evals. For analysis, we considered the Gibbs free energy binding (ΔG) results and the molecular interactions of the receptors with the ligands (complexed ligands, pyriproxyfen and the promising compounds).

### 3.9. Structure–Activity Relationship (SAR) and Molecular Overlay

Data on the structure–activity relationship (SAR) were collected according to the methodology proposed by Ferreira et al. (2019) [21], whereby a structural search of the promising compounds was carried out in the Scifinder^®^ (https://scifinder.cas.org/, accessed on 5 August 2020) database in order to obtain information related to experimental data, patents or research carried out with such molecules [72].

The molecular overlay was conducted according to the methodology proposed by da Silva Costa et al. (2018) [20]. Two or more three-dimensional chemical structures were superimposed, taking into account the contributions (%) of steric and electronic fields. The analyses were performed using Biovia Discovery Studio Visualizer (Dassault Systémes, Vélizy-Villacoublay, France) [59] software, considering the contributions of 50%, 70% and 100% of the steric field.

### 3.10. Molecular Dynamics (MD) Simulations 

GROMACS 5.1.4 (University of Groningen, Groningen, The Netherlands) software [37,38,40,41,42,43], available from the National Center for High-Performance Computing in São Paulo (CENAPAD-SP), was used for molecular dynamics (MD) simulations. The following parameters were used: time = 100 ns, 1 atm, 298 K, pH 6.0, GROMOS54A7 force-field updated [73], electrostatic treatment of PME [74], 1.0 nm for non-covalent interactions, periodic boundary conditions (PBC), 1 ps writing steps and SPC/E [75] with periodic boundary conditions (PBC) in a dodecahedral simulation box. Na^+^ and Cl^−^ ions were added to maintain the physiological salt concentration (0.15 M) and to neutralize the residual system charge at pH = 6.0. At first, the system was energy-minimized (steepest descent/conjugate gradient) until forces reached ≤ 10 kJ·mol^−1^ and nm^−1^, followed by a.

Then, a pre-equilibrium simulation step (heavy atoms’ position restrained for 1 ns) was performed under T = 298 K and system pressure maintenance at 1 atm (NPT ensemble) with a V-rescale thermostat [76] and Berendsen barostat [77]. To better simulate biological conditions with pH = 6.0, all pka residues were determined by PROPKA 3.1 [78,79]; all acidic, basic and histidine residues were be charged, whereas HIS-137 was deprotonated. Unrestrained simulation was performed for 100 ns for all systems with the SETTLE [80] algorithm for solvent bonds and the LINCS [81] algorithm for other bonds. The topology coordinates of the crystallographic ligand (JHIII) and all other ligands (pyriproxyfen, MP-073 and MP-416) were built in Automated Topology Builder (ATB) version 3.0 server (http://compbio.biosci.uq.edu.au/atb/, accessed on 9 May 2021) [82,83,84].

#### MMPBSA Free Energy Calculation (g-MMPBSA)

In addition to molecular docking and molecular dynamics simulation studies, molecular mechanics/Poisson–Boltzmann surface area (MM-PBSA) were applied to determine the thermodynamical stability of the AagJHBP-LASSBio-1386 complex and to investigate the contribution of each residue of the binding pocket. The MM-PBSA were calculated with a script-based g_mmpbsa tool [85]. This method calculates the binding energy (∆Ebinding), which represents the average of two energetic terms: potential energy in the vacuum (ΔEMM) and the free solvation energy (∆Gsolvation), as described by Equation (3).

∆Ebinding = ∆EMM + ∆Gsolvation(3)

The molecular mechanic (MM) energy term (ΔEMM) is calculated based on electrostatic (ΔEelec) and van der Waals (ΔEvdW) interaction components according to the molecular mechanics force-field parameters [85]. The solvation energy is computed based on polar (ΔGpol), using the Poisson–Boltzmann (PB) equation [86,87,88], and nonpolar solvation energy (ΔGnonpol), estimated from the solvent-accessible surface area (SASA), including repulsive and attractive forces between solute and solvent that are generated by cavity formation and van der Waals interactions [85].To decompose the binding energy, ΔEMM, ΔGpol and ΔGnonpol were first separately calculated for each residue and then summed to obtain the contribution of each residue to the binding energy [85].

The energy components EMM, Gpol and Gnonpol of the AagJHBP (Apo) and AagJHBP-LASSBio-1386 complexes were calculated for 700 snapshots extracted every 0.1 ns from the production trajectories between 30 and 100 ns. EMM was calculated using the LJ and Coulomb potential. To calculate Gpol, a box was generated using the extreme coordinates of the molecular complex in each dimension. The box was then expanded in each dimension by 1.5-fold to obtain a coarse-grid box (cfac = 1.5). A finer grid box was then placed within the coarse grid box, extending 5 Å (fadd = 5) from the complex’s extreme coordinates in each direction. An ionic strength of 0.150 M NaCl with radii of 0.95 and 1.81 Å for sodium and chloride ions, respectively, was used for all Gpol calculations. The values for the vacuum (vdie), solvent (sdie) and solute (pdie) dielectric constants were taken as 1, 80 and 2, respectively. The solvent radius was set to 1.4 Å, and temperature was set to 303 K. The linear PB equation was solved using the APBS program [89,90,91,92]. Gnonpolar was calculated using solvent-accessible surface area (SASA) nonpolar models using the surface tension (gamma) 0.0226778 KJ/(mol A^2^) and probe radius of 1.4 Å.

### 3.11. Synthetic Accessibility and Theoretical Synthetic Route of Promising Compounds

Synthetic accessibility (SA) is an important factor in the selection of potential bioactive compounds [52]. The SwissADME web server (http://www.swissadme.ch/, accessed on 5 June 2021) performs fragment-based SA prediction [93] by analyzing more than 13 million compounds delivered directly by vendors. This method takes into account the fact that frequent fragments imply high SA, i.e., easy synthesis, and rare fragments imply low SA, i.e., difficult synthesis. SA scores range from 1 (very easy) to 10 (very difficult).

### 3.12. Lipophilicity and Water Solubility via SwissADME Webserver

The main objective of this analysis was to establish solubility data of promising compounds for future in vivo assays, considering that the dilutions, preparation of solutions and types of chemical solvents used in these assays are an important with respect to obtaining accurate results. The partition coefficient between n-octanol and water (LogP_o/w_) is the main descriptor of lipophilicity. This physicochemical property plays an important role in the discovery of new bioactive compounds, in addition to being a very useful descriptor for pharmacokinetic analysis and prediction of the solubility of molecules [94,95]. Several methods can be used to estimate LogP_o/w_ for different types of chemical groups. The advantage of using multiple lipophilicity prediction methods is that these predictions can be optimized by opting for the most accurate method or by performing a consensual analysis between the methods. Thus, the more diverse the prediction methods, the more accurate the consensus value of LogP_o/w_ [96]. 

The SwissADME web server (http://www.swissadme.ch/, accessed on 5 June 2021) provides five prediction methods that can be used to obtain accurate data on promising compounds, with a view to future biological assays. XLOGP3 is an atomic predictive method with corrective factors that uses the LogP value of reference compounds as a starting point [97]. WLOGP is another atomistic method but without corrective factors, following the fragmentation method proposed by Wildman and Crippen [98]. MLOGP is a topological method based on the structure–LogP relationship of molecules of the most varied structures (from drugs to agrochemicals) using multiple linear regression analysis of 13 molecular descriptors associated with lipophilicity [99]. SILICOS-IT is a hybrid method that uses 27 fragments and 7 topological descriptors calculated using FILTER-IT (Silicos Co., Antwerp, Belgium) software (http://www.silicos-it.be/software.html, accessed on 5 June 2021). iLOGP is a recent physics-based method that uses the free energy of the solvation of n-octanol and water calculated using Born’s generalized implicit solvent equation and the solvent-accessible surface area (GB/SA) [100].

The solubility of a molecule in water (LogS) is an important characteristic used to define the form of dilution of a compound to determine its appropriate administration [101]. SwissADME provides three topological methods to determine water solubility: the ESOL method [102], the Ali method [103] and the SILICOS-IT method (http://www.silicos-it.be/software.html, accessed on 5 June 2021).

## 4. Conclusions

In this work, computational strategies for virtual screening based on ligands and pharmacophores were applied to search for new compounds with insecticidal activity against *Aedes aegypti*. The pharmacophoric hypotheses predicted the spatial characteristics of the ligands used in the alignment, along with the filters of physicochemical properties. This factor influenced the interaction of molecules obtained from these hypotheses with their respective biological targets for investigation in future studies. The results obtained from the pharmacokinetic, toxicological and biological activity predictions were satisfactory, considering that these results were fundamental to the selection of potential compounds.

Docking and molecular dynamics studies were fundamental for the selection of the promising molecules MP-416 and MP-073 based on the binding affinity values and the interactions with the amino acid residues present in the molecular targets used in this study. The analysis of the lipophilicity and solubility in water, as well as elaboration of the theoretical synthetic route, of the promising compounds will be essential for future biocidal tests for the validation of computational methods.

## Figures and Tables

**Table 1:** LD_50_ values of n-acyl piperidine compounds.

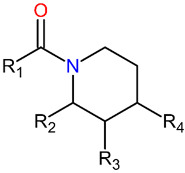			
**N**	**Substituents (R^1^, R^2^, R^3^ and R^4^)**	**LD_50_ (mM) ^[a]^**	**pLD**_**50**_^**[b]**^
**1**	R^1^: -(CH_2_)_6_CH_3_; R^3^: -CH_2_C_6_H_5_ (pivot molecule)	10.23	4.99
**2**	R^1^: -CH_2_C_6_H_11_; R^4^: -CH_2_C_6_H_5_	15.49	4.81
**3**	R^1^: -(CH_2_)_8_CHCH_2_; R^4^: -CH_2_C_6_H_5_	23.44	4.63
**4**	R^1^: -(CH_2_)_2_C_6_H_11_; R^4^: -CH_2_C_6_H_5_	24.55	4.61
**5**	R^1^: -(CH_2_)_10_CH_3_; R^2^: -CH_3_	32.36	4.49
**6**	R^1^: -CH_2_O_2_C_6_H_5_(CH)_3_	35.48	4.45
**7**	R^1^: -(CH_2_)_10_CH_3_; R^4^: -CH_3_	41.69	4.38
**8**	R^1^: -(CH_2_)_8_CHCH_2_; R^4^: -CH_2_C_6_H_5_	45.71	4.34
**9**	R^1^: -(CH_2_)_8_CH_3_; R^4^: -CH_3_	51.29	4.29
**10**	R^1^: -CH_2_C_6_H_10_CH_3_; R^3^: -CH_3_	57.54	4.24
**11**	R^1^: -(CH_2_)_3_C_6_H_11_; R^4^: -CH_3_	58.88	4.23
**12**	R^1^: -C_6_H_11_; R^4^: -CH_3_	79.43	4.10
**13**	R^1^: -(CH_2_)_8_CH_3_; R^2^: -CH_3_	93.33	4.03
**14**	R^1^: -(CH_2_)_8_CHCH_2_; R^2^: -CH_2_C_6_H_5_	95.50	4.02
**15**	R^1^: -(CH_2_)_8_CHCH_2_; R^4^: -CH_3_	97.72	4.01
**16**	R^1^: -(CH_2_)_5_CH_3_; R^3^: -CH_3_	102.33	3.99
**17**	R^1^: -(CH_2_)_2_C_6_H_11_; R^3^: -CH_3_	123.03	3.91
**18**	R^1^: -CH_2_C_6_H_11_; R^3^: -CH_3_	123.03	3.91
**19**	R^1^: -CH_2_C_6_H_11_; R^2^: -CH_3_	125.89	3.90
**20**	R^1^: -(CH_2_)_2_C_5_H_9_; R^2^: -CH_2_CH_3_	128.82	3.89
**21**	R^1^: -(CH_2_)_8_CHCH_2_; R^3^: -CH_3_	128.82	3.89
**22**	R^1^: -(CH_2_)_5_CH_3_; R^2^: -CH_3_	177.83	3.75
**23**	R^1^: -(CH_2_)_8_CHCH_2_; R^4^: -CH_2_CH_3_	181.97	3.74
**24**	R^1^: -(CH_2_)_8_CHCH_2_; R^2^: -CH_3_	190.55	3.72
**25**	R^1^: -(CH_2_)_2_C_6_H_11_; R^4^: -CH_3_	194.98	3.71
**26**	R^1^: -C_6_H_10_CH_3_; R^2^: -CH_3_	199.53	3.70
**27**	R^1^: -(CH_2_)_8_CHCH_2_; R^3^: -CH_2_CH_3_	213.80	3.67
**28**	R^1^: -(CH_2_)_2_C_6_H_11_; R^2^: -CH_3_	218.78	3.66
**29**	R^1^: -(CH_2_)_2_C_6_H_11_; R^2^: -CH_2_CH_3_	269.15	3.57
**30**	R^1^: -(CH_2_)_7_CH_3_; R^2^: -CH_2_CH_3_	301.90	3.52

^[a]^ LD_50_ = mM/mosquito. ^[b]^ pLD_50_ = −logLD_50_.

**Table 2:** Pearson correlation values between pharmacophoric features related to biological activity (pLD_50_ = −logLD_50_) of n-acyl piperidine compounds.

Pharmacophoric Features	ATM	SF	ARO	HYD	ACC
**SF**	0.85				
**ARO**	0.33	0.05			
**HYD**	0.68	0.90	−0.36		
**ACC**	−0.24	−0.25	0.62	−0.59	
**pLD_50_**	**0.39**	**0.27**	**0.64**	**0.01**	**0.18**

**Table 3:** Steric and electrostatic molecular field overlaps analyzed by Discovery Studio for n-acyl piperidine derivatives with 100% similarity.

Structures	Steric Contribution (%)	Electrostatic Contribution (%)
15/21	0.927	0.936
13/30	0.905	0.939
23/27	0.904	0.977
3/8	0.760	0.726
19/26	0.902	0.951
17/25	0.899	0.910
20/28	0.871	0.973

**Table 4:** Pharmacophoric hypotheses used in the search for new compounds.

Hypothesis 1					
Pharmacophoric Properties	X	Y	Z	Radius (Å)	Compounds Obtained from MolPort^®^ Database
**Acc 1**	20.904	−3.916	−1.2852	0.5	761
**Hyd 1**	17.7439	−4.8853	−2.2365	1.0	
**Hyd 3**	15.2453	−4.4747	−2.3858	1.0	
**Hyd 4**	24.6374	−5.4326	−2.516	1.0	
**Hyd 5**	24.2432	−6.5995	−2.7644	1.0	
**Hypothesis 2**					
**Acc 1**	20.904	−3.916	1.2852	0.5	279
**Hyd 1**	17.7439	−4.8853	−2.2365	1.0	
**Hyd 2**	16.4929	−4.6804	−2.3077	1.0	
**Hyd 4**	24.6374	−5.4326	−2.516	1.0	
**Hyd 5**	24.2432	−6.5995	−2.7644	1.0	
**Hypothesis 3**					
**Acc 1**	20.904	−3.916	1.2852	0.5	187
**Hyd 2**	16.4929	−4.6804	−2.3077	1.0	
**Hyd 3**	15.2453	−4.4747	−2.3858	1.0	
**Hyd 4**	24.6374	−5.4326	−2.516	1.0	
**Hyd 5**	24.2432	−6.5995	−2.7644	1.0	
**Hypothesis 4**					
**Acc 1**	20.904	−3.916	1.2852	0.5	52
**Hyd 1**	17.7439	−4.8853	−2.2365	1.0	
**Hyd 2**	16.4929	−4.6804	−2.3077	1.0	
**Hyd 3**	15.2453	−4.4747	−2.3858	1.0	
**Hyd 5**	24.2432	−6.5995	−2.7644	1.0	
**Hypothesis 5**					
**Acc 1**	20.904	−3.916	1.2852	0.5	33
**Hyd 1**	17.7439	−4.8853	−2.2365	1.0	
**Hyd 2**	16.4929	−4.6804	−2.3077	1.0	
**Hyd 3**	15.2453	−4.4747	−2.3858	1.0	
**Hyd 4**	24.6374	−5.4326	−2.516	1.0	
			**Total**		**1312 compounds**

**Table 5:** Carcinogenicity prediction by Preadmet server, according to pharmacophore-based virtual screening of compounds obtained from the MolPort^®^ database.

Structure	Carcinogenicity	
	Mouse	Rat
Pivot	−	−
JHIII	+	+
Pyriproxyfen	+	+
MP-961	+	+
MP-779	+	+
MP-073	+	+
MP-897	+	+
MP-488	+	+
MP-416	+	+
MP-930	+	+
MP-557	+	+
MP-112	+	+
MP-020	+	+
MP-232	+	+
MP-290	+	+

“−“ (negative) = carcinogenic molecule; “+” (positive) = non-carcinogenic molecule.

**Table 6:** Pharmacokinetic prediction by Preadmet server, according to pharmacophore-based virtual screening of compounds obtained from the MolPort^®^ database.

Structure		Absorption Properties			Distribution Properties	
	HIA (%) ^[a]^	PCaco-2 ^[b]^	PMDCK ^[c]^	Pskin ^[d]^	PPB (%) ^[e]^	C_brain_/C_blood_^[f]^
Pivot	100	54.39	28.18	−1.069	100	6.079
JHIII	98.57	52.97	4.54	−0.957	99.18	0.995
Pyriproxyfen	100	29.19	28.39	−1.820	98.57	1.085
MP-961	98.31	32.78	8.95	−3.200	92.54	0.306
MP-779	98.28	22.98	30.46	−3.884	88.15	0.878
MP-073	98.32	22.47	46.90	−3.927	88.23	1.097
MP-897	98.32	22.58	11.52	−3.863	92.21	0.427
MP-488	98.28	22.98	30.46	−3.884	88.15	0.878
MP-416	100	53.89	0.12	−2.579	82.71	0.466
MP-930	98.26	24.07	6.86	−3.440	92.68	0.797
MP-557	98.26	22.51	46.00	−4.055	94.64	0.395
MP-112	98.58	30.42	108.57	−1.519	91.41	0.330
MP-020	98.26	23.81	16.40	−3.529	91.48	0.828
MP-232	98.14	55.07	0.21	−3.655	86.42	0.037
MP-290	98.14	54.91	4.62	−3.424	90.23	0.058

^[a]^ HIA% = percentage of human intestinal absorption; ^[b]^ PCaco-2 = permeability of differentiated cells of the intestinal epithelium Caco-2 (nm s^−1^); ^[c]^ PMDCK = Mandin–Darby canine kidney cell permeability (nm s^−1^); ^[d]^ Pskin = skin permeability (cm h^−1^); ^[e]^ PPB (%) = plasma protein binding percentage; ^[f]^ Cbrain/Cblood = penetration of the blood–brain barrier.

**Table 7:** Steric and electrostatic molecular field overlaps analyzed by Discovery Studio for the pivot structure, the JHIII ligand, the commercial compound pyriproxyfen, the promising compounds identified in the MolPort^®^ database.

Structure	Overlay			
Pivot ^[^*^]^	**Molecule**	**50% ^[a]^**	**70% ^[b]^**	**100% ^[c]^**
	**MP-779**	0.56	0.63	0.76
	**MP-073**	0.47	0.55	0.71
	**MP-416**	0.66	0.70	0.78
	**MP-112**	0.41	0.53	0.74
JHIII	**Overlay**			
	**Molecule**	**50% ^[a]^**	**70% ^[b]^**	**100% ^[c]^**
	**MP-779**	0.42	0.55	0.74
	**MP-073**	0.39	0.51	0.74
	**MP-416**	0.69	0.69	0.77
	**MP-112**	0.52	0.59	0.76
Pyriproxyfen	**Overlay**			
	**Molecule**	**50% ^[a]^**	**70% ^[b]^**	**100% ^[c]^**
	**MP-779**	0.56	0.59	0.71
	**MP-073**	0.46	0.52	0.76
	**MP-416**	0.57	0.62	0.74
	**MP-112**	0.38	0.48	0.72

^[a]^ 50% = 50% steric and electrostatic contribution, respectively; ^[b]^ 70% = 70% steric contribution; ^[c]^ 100% = 100% steric contribution; ^[^*^]^ most active molecule of the studied series (pLD_50_ = 4.99).

**Table 8:** Binding energies (ΔE_binding_) calculated with the g_mmpbsa tool for AagJHBP complexes.

AagJHBP Complex	ΔE_binding_ (KJ/mol)
Holo	−216.21 ± 0.97
Pyriproxyfen	−435.96 ± 2.06
MP-073	−124.42 ± 1.08
MP-416	−265.95 ± 1.32

**Table 9:** Predicted SA score of promising compounds.

Structure	SA Score ^[a]^
Pivot	2.60
JHIII	3.52
Pyriproxyfen	3.30
MP-073	3.85
MP-416	2.12

^[a]^ SA scores range from 1 (very easy) to 10 (very difficult).

**Table 10:** Coordinates of active sites of molecular targets.

Receptor	Ligand	Coordinates of Grid Center	Grid Box Size
Juvenile Hormone-Binding Protein (*Aedes aegypti)* (PDB ID: 5V13)	Methyl (2E,6E)-9-[(2R)-3,3-dimethyloxiran-2-yl]-3,7-dimethylnone-2,6-dienoate	X = 239.301 Y = −26.500 Z = 353.846	32x 22y 20z

## Data Availability

The data presented in this study are available within the article.

## Supplementary Materials

The following supporting information can be downloaded at: https://www.mdpi.com/article/10.3390/ijms23179927/s1.

## Abbreviations

ACC	Acceptor
Ach	Acetylcholine
AchE	Acetylcholinesterase enzyme
CAS	Chemical Abstract Service
CNPq	National Council for Scientific and Technological Development
DEREK	Deductive estimation of risk from existing knowledge
LD	Lethal dose
HBA	Hydrogen-bonding acceptor
HBD	Hydrogen-bonding donator
HCA	Hierarchical cluster analysis
HIA	Human intestinal absorption
HYD	Hydrophobic
IGR	Insect growth regulator
IRAC	Insecticide Resistance Action Committee
JH	Juvenile hormone
LBDD	Ligand-based drug design
LogP	Lipophilicity
LogS	Water solubility
MM+	Molecular mechanical force field
MNA	Multilevel neighborhoods of atoms
MW	Molecular weight
OBP	Odorant-binding protein
WHO	World Health Organization
PAHO	Pan-American Health Organization
PCA	Principal component analysis
PDB	Protein Data Bank
PPB	Plasma–protein binding
QSAR	Quantitative structure–activity relationship
NMR	Nuclear magnetic resonance
RMSD	Root-mean-square deviation
RotB	Rotatable bond
SAR	Structure–activity relationship
SBDD	Structure-based drug design
VS	Virtual screening
UNIFAP	Federal University of Amapá
MD	Molecular dynamics
AagJHBP	Aedes aegypti juvenile hormone-binding protein
RMSF	Root-mean-square fluctuation
