# Supplementary material for: Identification of Potential New Aedes aegypti Juvenile Hormone Inhibitors from N-Acyl Piperidine Derivatives: A Bioinformatics Approach
Source: Int J Mol Sci. 2022 Sep 1;23(17):9927. doi: 10.3390/ijms23179927 (PMC9456062; doi:10.3390/ijms23179927)
Supplement: Supplementary file 1 [file ijms-23-09927-s001.zip › ijms-1867716-supplementary.pdf]

International Journal of *Molecular Sciences*

MDPI

Article

Identification of Potential New *Aedes aegypti* Juvenile Hormone Inhibitors from N-Acyl Piperidine Derivatives: A Bioinformatics Approach

Lúcio R. Lima [1,2], Ruan S. Bastos [1,2], Elenilze F. B. Ferreira [2,3], Rozires P. Leão [1,2], Pedro H. F. Araújo [2], Samuel S. da R. Pita [4], Humberto F. De Freitas [4,5], José M. Espejo-Román [6], Edla L. V. S. Dos Santos [2], Ryan da S. Ramos [2], Williams J. C. Macêdo [2,7] and Cleydson B. R. Santos [1,2,*]

1 Graduate Program in Medicinal Chemistry and Molecular Modeling, Federal University of Pará, Belém, PA, 66075-110, Brazil.
2 Laboratory of Modeling and Computational Chemistry, Department of Biological and Health Sciences, Federal University of Amapá, Macapá, 68902-280, AP, Brazil.
3 Laboratory of Organic Chemistry and Biochemistry, University of the State of Amapá, Macapá, 68900-070, AP, Brazil.
4 Bioinformatics and Molecular Modeling Laboratory, Federal University of Bahia, Av. Barão de Jeremoabo, 147, Pharmacy College , Ondina, Salvador, 40170-115, BA, Brazil.
5 Health department, State University of Feira de Santana, Feira de Santana, 44036-900 BA, Brazil
6 Department of Pharmaceutical and Organic Chemistry, Faculty of Pharmacy, Campus of Cartuja, University of Granada, 18071, Granada, Spain.
7 Laboratory of Molecular Modeling and Simulation System, Federal Rural University of Amazônia, Rua João Pessoa, 121, Capanema, 68700-030 PA, Brazil.
* Correspondence: breno@unifap.br (C.B.R.S)

Citation: Lastname, F.; Lastname, F.; Lastname, F. Title. *Int. J. Mol. Sci.* **2021**, *22*, x. https://doi.org/10.3390/xxxxx

Academic Editor: Firstname Lastname

Received: date
Accepted: date
Published: date

Publisher's Note: MDPI stays neutral with regard to jurisdictional claims in published maps and institutional affiliations.

Copyright: © 2021 by the authors. Submitted for possible open access publication under the terms and conditions of the Creative Commons Attribution (CC BY) license (https://creativecommons.org/licenses/by/4.0/).

Supplementary Material

Table S1. Pharmacophoric Model description, spatial coordinates, and radius.
Table S2. Pharmacophoric discrimination for structures with pLD_{50} values
Table S3. Physicochemical properties used as maximum and minimum filters.
Table S4. Prediction and identification of toxicophoric groups and their respective LD_{50} values.
Table S5. Binding affinity and interactions of best ranked compounds with the *Aedes aegypti* Mosquito Juvenile Hormone-Binding Protein (AagJHBP, PDB ID 5V13).
Table S6. Lipophilicity prediction (LogPo/w) through the SwissADME webserver ([52], http://www.swissadme.ch/).
Table S7. Solubility prediction (LogS) in water through the SwissADME webserver ([52], http://www.swissadme.ch/).

Figure S1. N-acyl piperidine derivatives 2D structures.
Figure S2. Main structural differences between N-acyl piperidine derivatives. (a) 15/21; (b) 13/30; (c) 23/27; (d) 03/08; (e) 19/26; (f) 17/25; (g) 20/28.
Figure S3. Structural superposition of crystallographic (green) and better docked pose (red) of JHII.
Figure S4. Secondary structure evaluation for productive phase of *Aedes aegypti* Juvenile Hormone-Binding Protein (AagJHBP) complexes analyzed by DSSP 3.1.4 [39-41] module installed on GROMACS 5.1.4 [37,38]. From upper left corner to the right: apo, holo, Pyri, MP-073 and MP-416.

Table S1. Pharmacophoric Model description, spatial coordinates and radius.

Pharmacophoric Characteristics		Coordinates			
		x	y	z	Radius
Hydrogen Acceptor	Acc 1	20.904	-3.916	-1.2852	0.5
Hydrophobic	Hyd 1	17.7439	-4.8853	-2.2365	1.0
Hydrophobic	Hyd 2	16.4929	-4.6804	-2.3077	1.0

Hydrophobic	Hyd 3	15.2453	-4.4747	-2.3858	1.0
Hydrophobic	Hyd 4	24.6374	-5.4326	-2.516	1.0
Hydrophobic	Hyd 5	24.2432	-6.5995	-2.7644	1.0

Table S2. Pharmacophoric discrimination for structures with pLD_{50} values.

Structure	ATM	SF	ARO	HYD	ACC	pLD_{50}
**1**	53	12	1	10	1	4.99
**2**	51	12	1	10	1	4.81
**3**	60	14	1	12	1	4.63
**4**	54	13	1	11	1	4.61
**5**	55	14	0	13	1	4.49
**6**	40	10	2	5	3	4.45
**7**	55	15	0	14	1	4.38
**8**	60	14	1	12	1	4.34
**9**	49	13	0	12	1	4.29
**10**	44	13	0	12	1	4.24
**11**	47	13	0	12	1	4.23
**12**	38	9	0	8	1	4.10
**13**	49	12	0	11	1	4.03
**14**	60	13	1	11	1	4.02
**15**	50	13	0	12	1	4.01
**16**	40	10	0	9	1	3.99
**17**	44	12	0	11	1	3.91
**18**	41	11	0	10	1	3.91
**19**	41	10	0	9	1	3.90
**20**	44	11	0	10	1	3.89
**21**	50	13	0	12	1	3.89
**22**	40	9	0	8	1	3.75
**23**	53	14	0	13	1	3.74
**24**	50	12	0	11	1	3.72
**25**	44	12	0	11	1	3.71
**26**	41	10	0	9	1	3.70
**27**	53	14	0	13	1	3.67
**28**	44	11	0	10	1	3.66
**29**	47	12	0	11	1	3.57
**30**	49	12	0	11	1	3.52

Table S3. Physicochemical properties for structures with pLD_{50} values.

Structures	MW[a]	RotB[a]	LogP[b]	PSA[a]	NºRings[a]	HBA[a]	HBD[a]
**1**	301.47	9	5.70	20.31	2	1	0
**2**	299.45	5	4.98	20.31	3	1	0
**3**	341.53	12	6.70	20.31	2	1	0

**4**	313.48	6	5.25	20.31	3	1	0
**5**	281.48	11	6.38	20.31	1	1	0
**6**	285.34	4	3.33	38.78	3	3	0
**7**	281.48	11	6.29	20.31	1	1	0
**8**	341.53	12	6.70	20.31	2	1	0
**9**	253.42	9	5.28	20.31	1	1	0
**10**	237.38	3	3.62	20.31	2	1	0
**11**	251.41	5	4.33	20.31	2	1	0
**12**	209.33	2	3.10	20.31	2	1	0
**13**	253.42	9	5.37	20.31	1	1	0
**14**	341.53	12	6.82	20.31	2	1	0
**15**	265.43	10	5.27	20.31	1	1	0
**16**	211.34	6	3.77	20.31	1	1	0
**17**	237.38	4	3.83	20.31	2	1	0
**18**	223.35	3	3.55	20.31	2	1	0
**19**	223.35	3	3.64	20.31	2	1	0
**20**	237.38	5	3.76	20.31	2	1	0
**21**	265.43	10	5.27	20.31	1	1	0
**22**	211.34	6	3.85	20.31	1	1	0
**23**	279.46	11	5.95	20.31	1	1	0
**24**	265.43	10	5.36	20.31	1	1	0
**25**	237.38	4	3.83	20.31	2	1	0
**26**	223.35	2	2.96	20.31	2	1	0
**27**	279.46	11	5.77	20.31	1	1	0
**28**	237.38	4	3.91	20.31	2	1	0
**29**	251.41	5	4.45	20.31	2	1	0
**30**	253.42	9	5.40	20.31	1	1	0
**Minimum**	211.34	2	2.96	20.31	1	1	0
**Maximum**	341.53	12	6.82	38.78	3	3	0

[a] Protox II (https://tox-new.charite.de/protox_II/); [b] Molinspiration (https://www.molinspiration.com/); MW=molecular weight; RotB=rotatable bonds; LogP=lipophilicity or hydrophobicity; PSA=polar surface area; HBA=hydrogen bond acceptors; HBD=hydrogen bond donator.

Table S4. Toxicology analysis and LD_{50} values for compounds.

Structures	Toxicity Prediction Alert (in human, rat and mouse)	Toxicophoric Group	Toxicity Alert	Toxicity Class[a]	LD_{50}[b]
Pivot	Phospholipidosis	Etamine or piperidine	Plausible	4	1000
Pyriproxyfen	-	-	No alert	5	2830
		**Hypothesis 1**			
MP-961	-	-	No alert	5	2100
MP-779	-	-	No alert	6	5400
MP-073	-	-	No alert	6	20000
MP-897	-	-	No alert	6	20000
MP-488	-	-	No alert	6	5400
MP-416	-	-	No alert	4	955
MP-930	-	-	No alert	6	20000
MP-557	-	-	No alert	5	5000
MP-112	-	-	No alert	4	350
		**Hypothesis 3**			
MP-020	-	-	No alert	6	20000
MP-232	-	-	No alert	4	1000
MP-290	-	-	No alert	5	2500

[a]Class 1: fatal if ingested. ($LD_{50} \leq 5$); Class 2: fatal if ingested ($5 < LD_{50} \leq 50$); Class 3: toxic if ingested ($50 < LD_{50} \leq 300$); Class 4: dangerous if ingested ($300 < LD_{50} \leq 2000$); Class 5: can be dangerous if ingested ($2000 < LD_{50} \leq 5000$); Class 6: non toxic ($LD_{50} > 5000$). Data obtained from the Protox II online server (https://tox-new.charite.de/protox_II/). [b]LD_{50}= $mg.kg^{-1}$.

Table S5. Binding affinity and interactions of best ranked compounds with the *Aedes aegypti* Mosquito Juvenile Hormone-Binding Protein (AagJHBP, PDB ID 5V13).

Structures	ΔG[a]	Hydrogen Bonding (Distance)	Hydrophobic Interactions
JHIII (Complex ligand)	-7.53	**TYR129** (2.75 Å)	**TYR33**, **VAL34**, **VAL51**, **TRP53**, **PRO55**, **TYR64**, **VAL65**, **VAL68**, **TYR129**, **PHE144**, **PHE269**, **TRP278** and **ALA281**.
Pyriproxyfen (Commercial Compound)	-10.04	**TYR129** (2.22 Å)	**TYR33**, **VAL51**, **TRP53**, **PRO55**, **TYR64**, **VAL65**, **VAL68**, **TYR129**, TYR133, **PHE144** and **ALA281**.
MP-779	-6.21	-	**TYR33**, LEU37, TRP50, **VAL51**, **TRP53**, **PRO55**, **TYR64**, **VAL65**, **VAL68**, TYR133, GLY146, **PHE269**, **TRP278** and **ALA281**.
MP-073	-9.48	**TYR33** (3.47 Å)	**TYR33**, LEU37, **VAL51**, **TYR64**, **VAL68**, **TYR129**, TYR133 and **ALA281**.
MP-416	-10.06	-	LEU30, **TYR33**, **VAL34**, LEU37, TRP50, **VAL51**, **TRP53**, **PRO55**, **TYR64**, **VAL65**, **VAL68**, LEU72, LEU74, **TYR129**, TYR133, **PHE144**, **PHE269**, **TRP278** and **ALA281**.
MP-112	-7.36	**TRP53** (3.05 Å)	**TYR33**, **VAL34**, LEU37, TRP50, **VAL51**, **TRP53**, **PRO55**, TRP64, **VAL65**, **VAL68**, TYR133, **PHE269**, **TRP278** and **ALA281**.

[a] Binding energy of the best conformation (kcal. mol^{-1}).

Table S6. Lipophilicity prediction (LogPo/w) through the SwissADME webserver ([52], http://www.swissadme.ch/).

Molecule	iLOGP	XLOGP	WLOGP	MLOGP	SILICOS-IT	Consensus
**Pivot**	4.04	5.42	4.45	4.04	5.12	4.61
**Pyriproxyfen**	3.94	5.52	5.33	4.04	4.63	4.69
**MP-779**	2.95	3.35	2.87	3.67	3.58	3.28
**MP-073**	2.86	2.84	2.48	3.44	2.67	2.86
**MP-416**	3.82	4.21	3.78	3.39	4.20	3.88
**MP-112**	3.02	4.50	4.87	2.89	3.93	3.84

Table S7. Water solubility prediction (LogS) through the SwissADME webserver ([52], http://www.swissadme.ch/).

Molecule	ESOL	Ali	SILICOS-IT	Consensus
**Pivot**	-4.73	-5.60	-6.05	-5.46
**Pyriproxyfen**	-5.40	-5.86	-7.47	-6.24
**MP-779**	-3.98	-3.81	-4.30	-4.03
**MP-073**	-3.58	-3.28	-3.47	-3.44
**MP-416**	-4.42	-4.41	-6.21	-5.01
**MP-112**	-4.37	-5.01	-4.55	-4.64

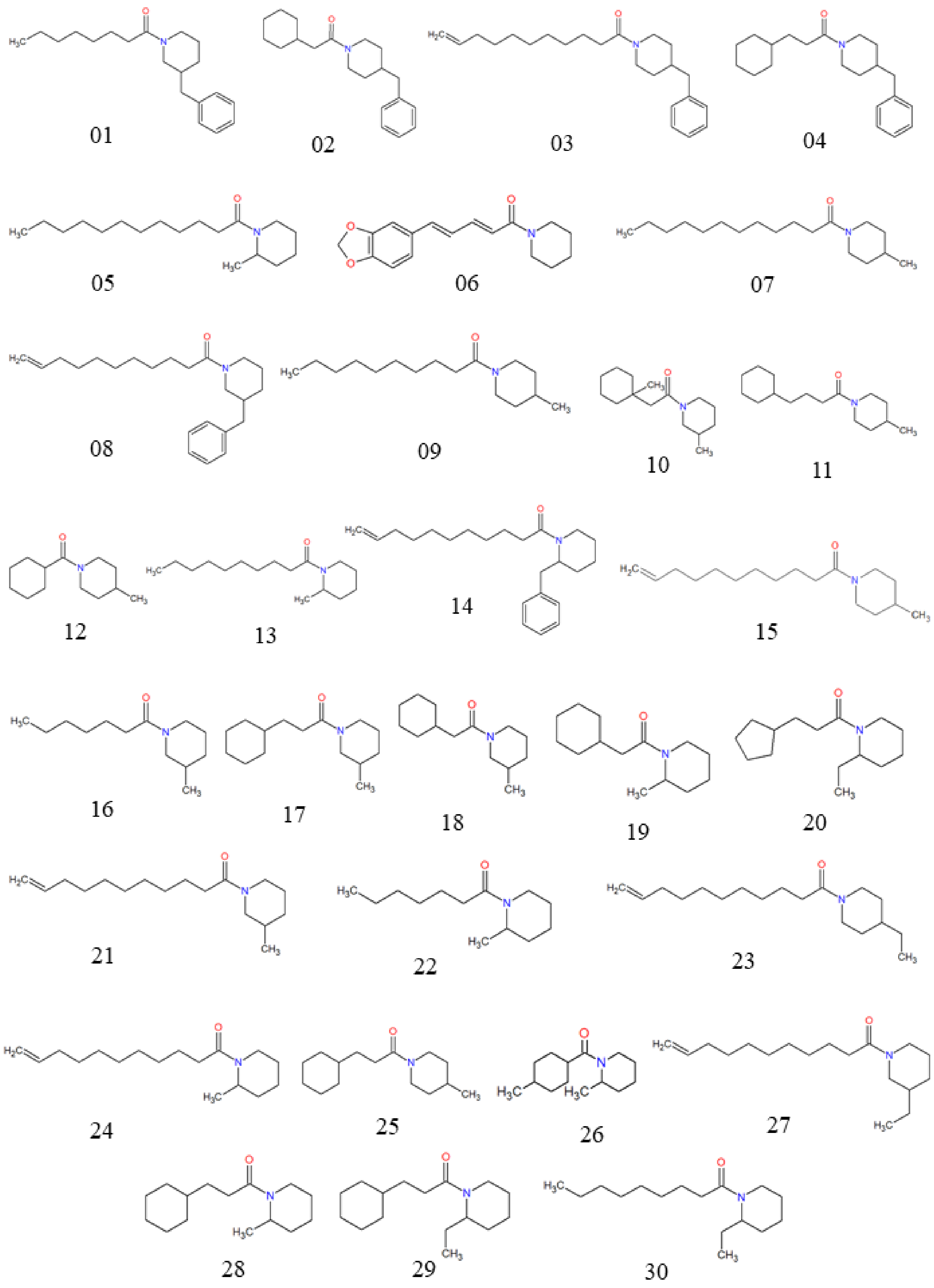

Figure S1. N-acyl piperidine derivatives 2D structures.

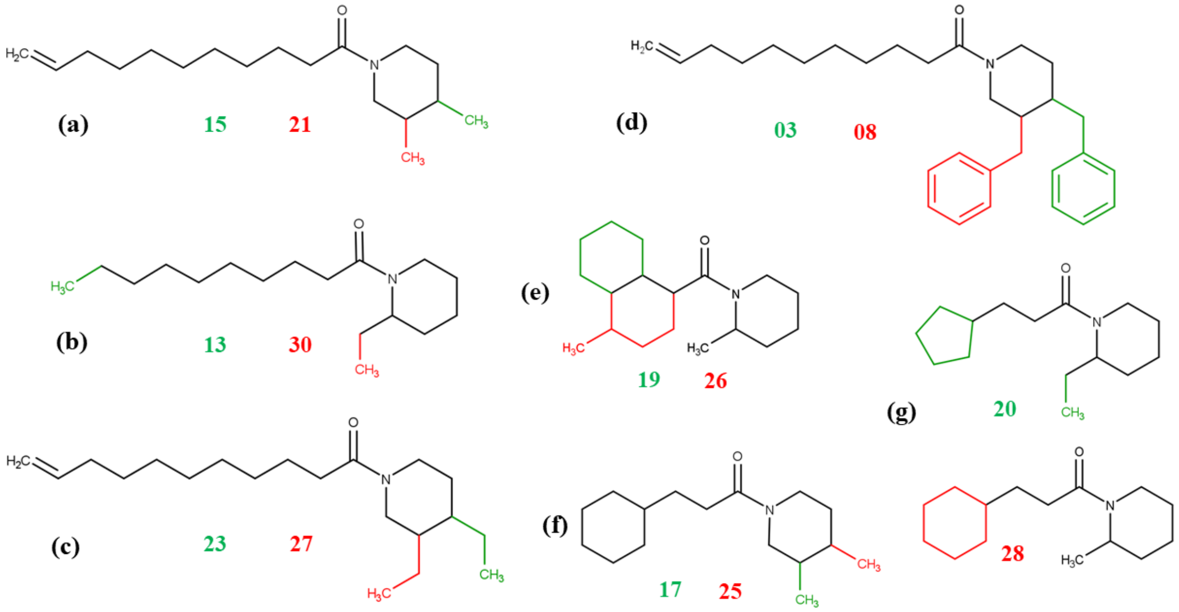

Figure S2. Main structural differences between N-acyl piperidine derivatives. (a) 15/21; (b) 13/30; (c) 23/27; (d) 03/08; (e) 19/26; (f) 17/25; (g) 20/28.

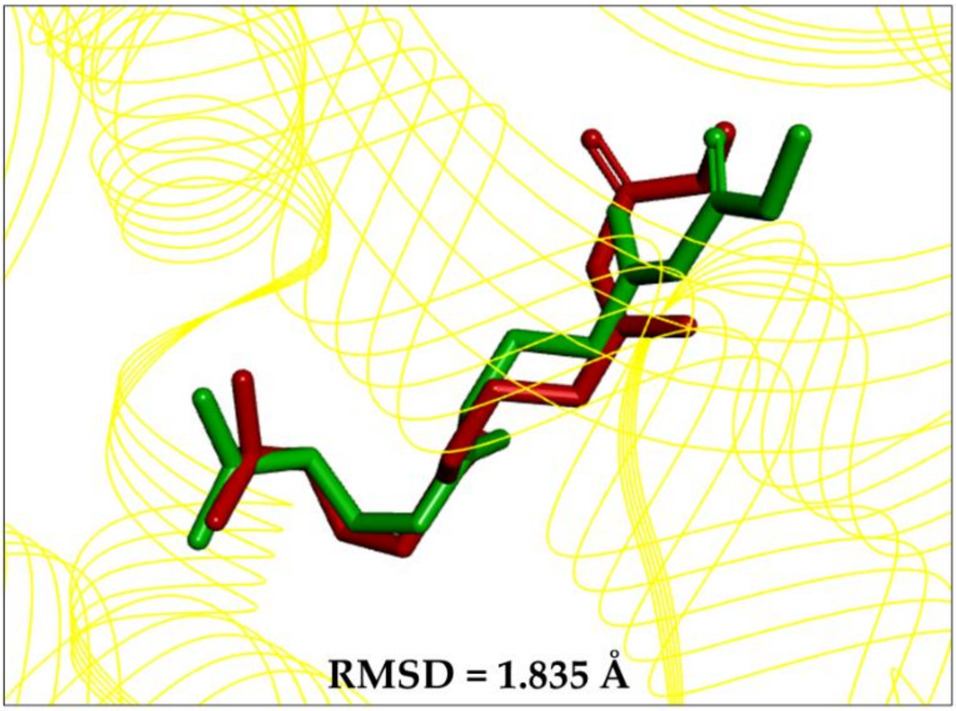

Figure S3. Structural superposition of crystallographic (green) and better docked pose (red) of JHII.

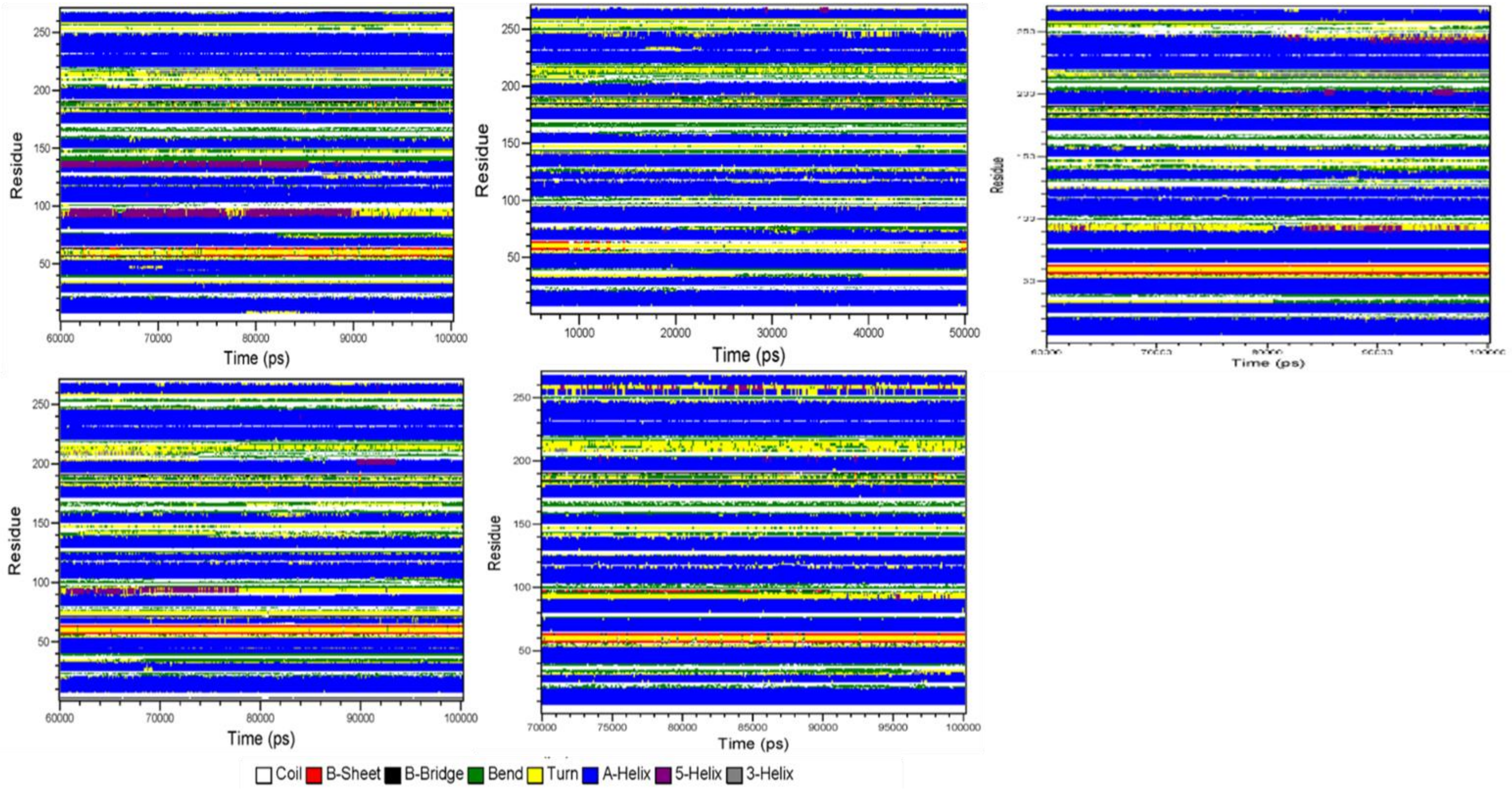

Figure S4. Secondary structure evaluation for productive phase of Aedes aegypti Juvenile Hormone-Binding Protein (AagJHBP) complexes analyzed by DSSP 3.1.4 [38-41] module installed on GROMACS 5.1.4 [37,38]. From upper left corner to the right: apo, holo, Pyri, MP-073 and MP-416.
